# Long non-coding RNA *BZRAP1-AS1* functions in malignancy and prognosis for non-small-cell lung cancer

**DOI:** 10.7717/peerj.13871

**Published:** 2022-08-23

**Authors:** Xuefeng Hao, Minghang Zhang, Meng Gu, Ziyu Wang, Shijie Zhou, Weiying Li, Shaofa Xu

**Affiliations:** 1 Department of Cancer Research Center, Beijing Chest Hospital, Capital Medical University/Beijing Tuberculosis and Thoracic Tumor Research Institute, Beijing, China; 2 Department of Thoracic Surgery, Beijing Chest Hospital, Capital Medical University/Beijing Tuberculosis and Thoracic Tumor Research Institute, Beijing, China

**Keywords:** Non-small-cell lung cancer, Long non-coding RNA, BZRAP1-AS1, Prognosis, Proliferation, Migration

## Abstract

**Purpose:**

The function of *BZRAP1-AS1* is unknown in lung cancer. We evaluated the clinicopathologic significance of *BZRAP1-AS1*, and its role in non-small-cell lung cancer (NSCLC) progression.

**Patient and methods:**

Sixty-three NSCLC patients from Beijing Chest Hospital were included. The expression of *BZRAP1-AS1* was detected by real-time quantitative polymerase chain reaction (RT-qPCR) in tumor tissues and adjacent normal tissues. Then, the clinicopathological significance and prognostic value of *BZRAP1-AS1* were analyzed by using our cohort and TCGA cohort. Finally, the effect of *BZRAP1-AS1* on proliferation and motility of NSCLC cell lines were evaluated by cell growth assay, colony formation assay, xenograft tumorigenesis experiment in nude mice and transwell assays respectively.

**Results:**

Compared with adjacent normal tissues, *BZRAP1-AS1* showed lower expression in NSCLC tumor tissues. As for the relationship between *BZRAP1-AS1* and clinical characteristics, our results were consistent with those of TCGA data. *BZRAP1-AS1* was lower in T1 than T2–T4 patients, N1-N3 than N0 patients. Low level *BZRAP1-AS1* was related to shorter overall survival time (OS) in lung adenocarcinoma (LUAD), and poor first progression time (FP) in LUAD and lung squamous cell carcinoma (LUSC) patients. *BZRAP1-AS1* was significantly associated with the prognosis of NSCLC patients. Overexpression of *BZRAP1-AS1* inhibited proliferation and migration of H1299 and HCC827 cells.

**Conclusion:**

*BZRAP1-AS1* expression decreases in tumor tissues with the increase of malignancy grades in NSCLC. *BZRAP1-AS1* plays an anticancer role by inhibiting cell proliferation, invasion, and metastasis, and has a potential prognostic value in NSCLC. *BZRAP1-AS1* may serve as a diagnostic marker and therapeutic target for NSCLC.

## Introduction

Lung cancer is one of the malignant tumors with the highest morbidity and mortality. There were 2.2 million new cases diagnosed and 1.8 million deaths worldwide in 2020, as the latest Global Cancer Statistics has shown (Sung et al., 2021). In lung cancer, NSCLC accounts for about 80%, and with 60% lung adenocarcinoma (LUAD) and 25% lung squamous cell carcinoma (LUSC) as the predominant pathologic subtypes (Zheng, 2016). Although multiple therapy regimens including surgery, chemotherapy, radiotherapy, immunotherapy are used, the prognosis of NSCLC patients is still poor. Therefore, it is very urgent to explore the molecular mechanism of the occurrence and development of lung cancer and the search for new therapeutic targets. Recent evidences have shown that lncRNAs have important functions in regulation of gene expression, RNA splicing, and nucleation of subnuclear domains (Statello et al., 2021). Many lncRNAs, such as HOTAIR, PVT1, LINC01123 have been implicated in development or progression of lung cancer (Loewen et al., 2014; Liu et al., 2018; Zheng et al., 2020a; Pan et al., 2020; Xi et al., 2020; Hua et al., 2019).

As a novel lncRNA, benzodiazapine receptor associated protein 1 antisense RNA 1 (*BZRAP1-AS1*), also named TSPOAP1-AS1, was mentioned firstly in Alzheimer’s disease (Jun et al., 2017; Witoelar et al., 2018; Tan et al., 2021a). Subsequently, studies indicated that BZRAP1-AS expression was dysregulated in several cancers and played critical roles in tumor initiation and progression  (Giulietti et al., 2018; Tan, Jin & Wang, 2019; Wang et al., 2019a; Wang et al., 2019b; Zheng et al., 2020b; Tang et al., 2021). However, whether *BZRAP1-AS1* as oncogene or tumor suppressor is still unclear in cancers. For example, knocking down *BZRAP1-AS1* in hepatocellular carcinoma (HCC) cells inhibits HUVEC proliferation, migration, and angiogenesis, the lncRNA tends to be an oncogene (Wang et al., 2019b); however, the conclusions are opposite in cervical cancer, prostate cancer, and lung adenocarcinoma (Tan, Jin & Wang, 2019; Zheng et al., 2020b; Tang et al., 2021). The study of BZRAP1-AS1 in lung adenocarcinoma is only from prognostic bioinformatics analysis studies have been reported, the design is very limited only through bioinformatics to study lung adenocarcinoma (Tang et al., 2021). In this study, 63 NSCLC cases were collected and *BZRAP1-AS1* levels in tumor and adjacent normal tissues were examined. Then the association between *BZRAP1-AS1* and clinical parameters was analyzed using 63 NSCLC cases cohort and TCGA database. Finally, the effect of *BZRAP1-AS1* on cell proliferation and migration was investigated in *vivo* and in *vitro*.

## Patients and Methods

### Patient samples

The samples were collected as previously described in Zhang et al. (2021) (28 April 2021). After calculation, a sample size of at least 41 cases is required based on the pre-experimental data (95% confidence interval, 80% statistical power). Specifically, 63 NSCLC patients (46 males and 17 females; mean age of 61.83  ± 9.29 years) were enrolled between 2018-10-24 and 2020-12-22. All above patients received surgical treatment and no preoperative neoadjuvant therapy at Beijing Chest Hospital. Tumor tissues and adjacent normal tissues were obtained. All patients had no other accompanying malignancy. After resection, tissue samples were flash frozen in liquid nitrogen for 30 min within 2 h and then frozen at −80 °C for long-term storage. This study was approved by the Ethics Committee of Beijing Chest Hospital. Ethical Approval Number: 2019-71.

### Cell lines and culture conditions

Cell culture and part assay methods were performed as previously described (Zhang et al., 2021). Human NSCLC cell lines H226 were obtained from the National Institutes of Health (NIH). The human NSCLC cell lines A549, H1395, H1299, HCC827 and H1703 were obtained from the National Infrastructure of Cell Line Resource (NICR). All cell lines were cultured in RPMI-1640 medium (Invitrogen, Carlsbad, CA, USA) supplemented with 10% fetal bovine serum (FBS; Gibco, Los Angeles, CA, USA). The cells were maintained at 37 °C in a humidified chamber containing 5% CO2.

### RNA extraction and Quantitative Real-Time PCR (RT-qPCR)

Total RNA was extracted from stored tissues and cells with the TRIzol reagent (Ambion, Carlsbad, CA, USA), according to the manufacturer’s instructions. NanoDrop 2000c (Thermo Scientific, Waltham, MA, USA) was used to measure the concentration of RNA. RNAs were reverse-transcribed into cDNA in a 20 µl system using the TransScrip First-strand cDNA Synthesis SuperMix (TRAN) according to the protocol. The quantitative PCR was performed according to the instructions of the SYBR®. Applied Biosystems 7500 Fast Real-Time PCR System (Thermo Fisher Scientific) was used for qPCR with a 15-µl reaction system, including 7.5 µl of PowerUp™ SYBR™ Green Master Mix (applied biosystems), 2.5 µl of RNase-free water, 1.25 µl of upstream and 1.25 µl of downstream primers, and 2.5 µl of cDNA template. GAPDH was used as an internal reference gene. The primer sequences involved as follows. *BZRAP1-AS1* (F): TGTCTGCATCCCACAACAGG, (R): GGACCAGCTTGGAGTTGTGT. GAPDH (F): ACTAGGCGCTCACTGTTCTC, (R): CGACCAAATCCGTTGACTCC (5′–3′).

### Lentivirus construction and infection

The lentiviruses for *BZRAP1-AS1* overexpression (OE) (Lv-*BZRAP1-AS1*, 66037-1) and negative control (NC) (LVCON238) both were purchased from the Shanghai Genechem Company. Lentivirus infection was performed in H1299 and HCC827 NSCLC cells following the manufacturer’s instruction. Firstly, H1299 and HCC827 cells were plated in 96-well culture plates at a density of 3–5 × 10^3^/well. The next day, cells were infected with Lv-*BZRAP1-AS1* and LVCON238 for 12 h. The infection efficiency was about 80%, 48–72 h after infection. Finally, the infected cells were screened with Puromycin (2 µg/mL) for 2–3 days.

### Online database analysis

Gene Expression Profiling Interactive Analysis (GEPIA) (http://gepia.cancer-pku.cn/index.html) is an online tool for gene expression analysis based on TCGA and GTEx data (Tang et al., 2017). In this study, the expression of *BZRAP1-AS1* in multiple tumor samples and paired normal tissues was evaluated using GEPIA. Transcriptome profiling HTSeq-Counts data and clinical information of NSCLC patients were downloaded from the TCGA database (https://portal.gdc.cancer.gov). The expression of BZRAP1-AS in Lung normal tissues and cancer adjacent normal tissues were both downloaded from the GTEx database. Then the expression difference and OS survival analysis of *BZRAP1-AS1* in NSCLC was analyzed by SPSS and GraphPad Prism. Additionally, FP and univariate survival analysis was made by KM Plotter, which is an online survival analysis tool based on data from GEO, EGA, and TCGA (Győrffy et al., 2013; Lánczky & Győrffy, 2021).

### Cell growth curve assay

H1299 cells (4 ×10^3^/well) and HCC827 cell (3 ×10^3^/well) were seeded in 96-well plates. Five repetitive wells were set for each experiment. Cell activity was tested by cell counting kit-8 (CCK-8) (Dojindo, Kyushu, Japan) for 4 days. 10 µl CCK-8 was added into each well and then incubated with the cells for 2 h at 37 °C. The absorbance was recorded at 450nm with Bio Tek Epoch Microplate Reader (Bio Tek Instruments, Inc). The experiments were repeated 3 times.

### Colony formation assay

300 cells/well were plated into 6-well plates and routinely cultured for 7–14 days. Subsequently the cells were washed quickly with PBS buffer solution and stained with 0.2% gentian violet. The number of colonies (≥50 cells) was recorded under an optical microscope. The experiments were repeated three times.

### Tumor growth in nude mice

All animal experiments were approved by the Ethics Committee of the Beijing Chest Hospital, Capital Medical University, Beijing, China (No. 2021-059), in compliance with national or institutional guidelines for the care and use of animals. Female BALB/c-nu mice (aged 4–6 weeks) were purchased from Beijing Vital River Laboratory Animal Technology Co., Ltd. The average weight of mice was 17 g. All mice were housed in animal facility under pathogen-free conditions. The animal room was kept at 18–23 °C, 40%–60% humidity, and a 10 h–14 h light-dark cycle. In order to get the most scientific experimental results, at least 10 mice in each group, but according to the 3R-reduction principle, so six mice in each group, a total of 24 mice were randomly divided into four groups. All food, cages, water, and other items that contact mice were sterile and handled using aseptic technique within a certified biosafety cabinet. Cells (H1299 NC *vs* H1299 OE, HCC827 NC *vs* HCC827 OE) were collected and resuspended in 10% RMPI-1640 at a density of 1  × 10^7^ cells/ml. 2 × 10^6^ were injected into subcutaneously the right armpit of the mice, NC as control group. Then length (L) and width (W) sizes of tumors were measured regularly every 2–3 days by researchers who didn’t know the grouping. The mice were euthanized on the 30–40th day, and tumors were harvested and photographed. Tumor volume was analyzed by *V* = 1/2(*L* × *W*^2^). On the 40th day following tumor injection and the average tumor diameter does not exceed 20 mm, or if ulceration, infection, or necrosis occurs, the experiment was terminated and the mice were euthanized using a standard carbon dioxide method. Finally, tumors were harvested and weighed. Data was analyzed using GraphPad Prism, the tumor volume data was analyzed using ANOVA and weight differences by unpaired *t*-test.

### Cell migration and invasion assays

Cell invasion was tested using Transwell chamber (8 µm in pore size, Costar, Beijing, China) pre-coated with Matrigel (Corning Matrigel Matrix, CA, USA). Cells were cultured in serum-free RMPI-1640 medium for 24 h. HCC827 cells (2  × 10^6^) and H1299 cells (1.5  × 10^6^) were seeded in the upper compartment with serum free medium. The lower chamber was filled with RMPI-1640 medium containing 10% FBS. After 48 h, the chamber was taken out and stained with 0.2% gentian violet for 20min, then upper Matrigel was washed away the staining reagent and removed using cotton swab cautiously. Finally, metastatic cells were observed and pictures taken under a Nikon microscope. The experiments were repeated three times.

### RNA-seq data analysis of mice tumor tissues and co-expression analysis

Subcutaneous tumors were RNA sequenced in H1299 NC group and H1299 *BZRAP1-AS1* OE group. And different genes between the two groups were screened using EdgeR, then performed GO (Gene Ontology) enrichment analysis and KEGG (Kyoto Encyclopedia of Genes and Genomes) pathway enrichment analysis for up-regulated genes and down-regulated genes. Then, we selected genes with significant differences in expression and closely related to tumor proliferation, metastasis and signaling pathways, and predicted the genes affected by *BZRAP1-AS1* based on the above results. Furthermore, the co-expression analysis between *BZRAP1-AS1* and predicted genes was performed using Starbase Analysis platform (https://starbase.sysu.edu.cn/) (Li et al., 2014).

### Statistical analysis

The experiments were repeated three times. Normal distribution data were presented as Mean  ± SD, and compared using a two-tailed *t*-test. Abnormal distribution data were presented as median (25–75 percentile), and compared using the nonparametric test. The log-rank test was used to compare Kaplan–Meier survival curves. Statistical methods used for RNA-Seq analysis and TCGA data analysis were described above. *P* < 0.05 was considered statistically significant.

## Results

### *BZRAP1-AS1* expression reduced in NSCLC tumor tissues

**Figure 1:**
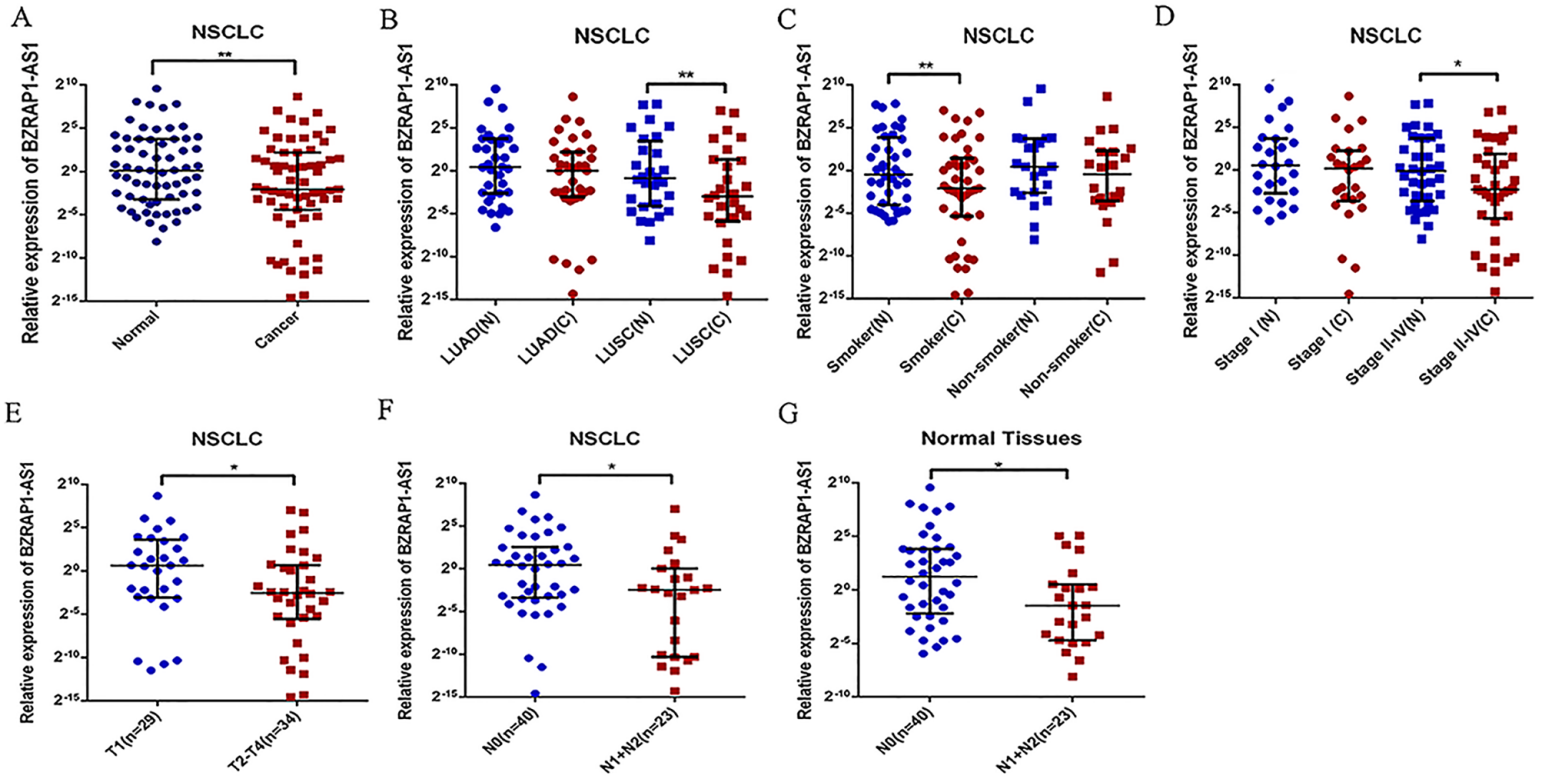
Relative *BZRAP1-AS1* expression in cancer and normal tissues detected by RT-qPCR. Relative *BZRAP1-AS1* expression in 63 pairs NSCLC cancer (C) and normal (N) tissues from Beijing Chest Hospital detected by RT-qPCR. (A) Relative *BZRAP1-AS1* in cancer and normal tissues. (B) Relative *BZRAP1-AS1* expression in cancer and normal tissues of different histologic types. (C) Relative *BZRAP1-AS1* expression in cancer and normal tissues of nonsmokers and smokers in own samples. (D) Relative *BZRAP1-AS1* expression in the NSCLC TNM stage I and stage II-IV groups. (E) Relative *BZRAP1-AS1* expression in the NSCLC stage T1 and T2-T4 groups. (F–G) Relative *BZRAP1-AS1* expression in stage N0 and N1 + N2 groups. *Statistical significance *P* < 0.05, **Statistical significance *P* < 0.01 in own samples.

The results showed that the expression of *BZRAP1-AS1* was significantly lower in tumor tissues than in adjacent normal tissues (Table 1, Fig. 1A). The nonparametric test was performed. The median was 0.240 (25–75 percentile, 0.046–4.556) in tumor tissues and 1.076 (25–75 percentile, 0.106–13.380) in adjacent normal tissues (*P* = 0.004). Then, further subgroup analysis exhibited that in >60 years old [0.228 (0.487–2.486) *vs* 1.048 (0.143–14.076), *P* = 0.016], male [0.226 (0.027–3.267) *vs* 0.821 (0.097–12.904), *P* = 0.013], smoker [0.235 (0.025–2.695) *vs* 0.737(0.061–14.324), *P* = 0.007], LUSC [0.126 (0.017–2.486) *vs* 0.556 (0.059–11.459), *P* = 0.002], and stage II–IV subgroup [0.205 (0.019–3.697) *vs* 0.905 (0.081–13.452), *P* = 0.012], *BZRAP1-AS1* was lower in tumor tissues than adjacent normal tissues. There was no expression difference in ≤60 years old, female, non-smokers, LUAD, and stage I subgroup (Table 1, Figs. 1B–1D).

**Table 1:** Relative *BZRAP1-AS1* expression in cancer and normal tissues.

**Group**	**No. of cases**	***BZRAP1-AS1* expression (Median, 25–75 Percentile)**		***P* value**
		**Tumor tissues**	**Normal tissues**	
Total	63	0.240,0.046–4.556	1.076,0.106–13.380	**0.004**
Ages				
≤60	31	0.293,0.271–5.645	1.076, 0.528–9.019	0.092
>60	32	0.228,0.487–2.486	1.048, 0.143–14.076	**0.016**
Sex				
Male	46	0.226, 0.027–3.267	0.821, 0.097–12.904	**0.013**
Female	17	1.261, 0.100–5.245	1.763, 0.130–13.620	0.124
Smoking history				
No	22	0.751,0.086–4.913	1.380,0.165–13.416	0.236
Yes	41	0.235,0.025–2.695	0.737,0.061–14.324	**0.007**
Histologic type				
LUAD	35	1.020,0.123–4.581	1.413,0.167–13.380	0.174
LUSC	28	0.126,0.017–2.486	0.556,0.059–11.459	**0.002**
TNM stage				
I	26	1.128,0.081–4.847	1.467,0.153–12.988	0.096
II–IV	37	0.205,0.019–3.697	0.905,0.081–13.452	**0.012**
T stage				
T1	29	1.528,0.117–12.300	1.763, 0.264–17.199	0.090
T2–T4	34	0.169, 0.021–1.594	0.364, 0.066–6.737	**0.007**
N stage				
N0	40	1.354, 0.094–5.843	2.346, 0.216–14.076	**0.011**
N1 + N2	23	0.180, 0.001–1.020	0.359, 0.038–1.413	0.153

**Notes.**

Bold indicates that the difference is statistically significant (*P* < 0.05).

### Relation between the expression of *BZRAP1-AS1* and the clinical characteristics of NSCLC

To find the potential biological functions of *BZRAP1-AS1*, the relation between clinical characteristics and *BZRAP1-AS1* in tumor and adjacent normal tissues was analyzed respectively (Table 2). In tumor tissues, *BZRAP1-AS1* was lower in T2–T4 than T1 patients [0.169 (0.021–1.594) *vs* 1.528 (0.117–12.300), *P* = 0.040], and N1-N2 than N0 patients [0.180 (0.001–1.020) *vs* 1.354 (0.094–5.843), *P* = 0.032] (Table 2, Figs. 1E–1F). And in adjacent normal tissues, *BZRAP1-AS1* was lower in N1+N2 than N0 patients (0.359 (0.038–1.413) *vs* 2.346 (0.216–14.076), *P* = 0.018). Whether tumor tissues or adjacent normal tissues was no difference in subgroups divided by smoking history, histologic type, pleura invasion, and vessel carcinoma embolus. Additionally, the analysis results in subgroups may be biased because of the limitation of sample size, which still need to be validated.

**Table 2:** Clinicopathological characteristics of NSCLC patients and expression of *BZRAP1-AS1* in tumor tissues and normal tissues.

**Variables**	**No. of Cases**	***BZRAP1-AS1* expression (Median, 25–75 Percentile)**			
		**Tumor tissues**	*P* value	**Normal tissues**	***P* value**
Smoking history			0.535		0.604
No	22	0.751, 0.086–4.913		1.380, 0.165–13.416	
Yes	41	0.235, 0.025–2.695		0.737, 0.061–14.324	
Histologic type			0.103		0.326
LUAD	35	1.020, 0.123–4.581		1.413, 0.167–13.380	
LUSC	28	0.126, 0.017–2.486		0.556, 0.059–11.459	
T stage			**0.040**		0.071
T1	29	1.528, 0.117–12.300		1.763, 0.264–17.199	
T2–T4	34	0.169, 0.021–1.594		0.364, 0.066–6.737	
Lymphatic metastasis			**0.032**		**0.018**
N0	40	1.354, 0.094–5.843		2.346, 0.216–14.076	
N1 + N2	23	0.180, 0.001–1.020		0.359, 0.038–1.413	
Pleura invasion			0.333		0.956
Negative	35	0.185, 0.046–1.564		0.737, 0.135–13.525	
Positive	28	1.408, 0.048–5.379		1.145, 0.053–12.290	
Vessel carcinoma embolus			0.596		0.845
Negative	37	0.235, 0.037–3.696		0.905, 0.120–10.858	
Positive	26	0.630, 0.082–6.116		1.133, 0.077–14.382	

**Notes.**

Bold indicates that the difference is statistically significant (*P* < 0.05).

### *BZRAP1-AS1* expression and clinical parameters in TCGA

**Figure 2:**
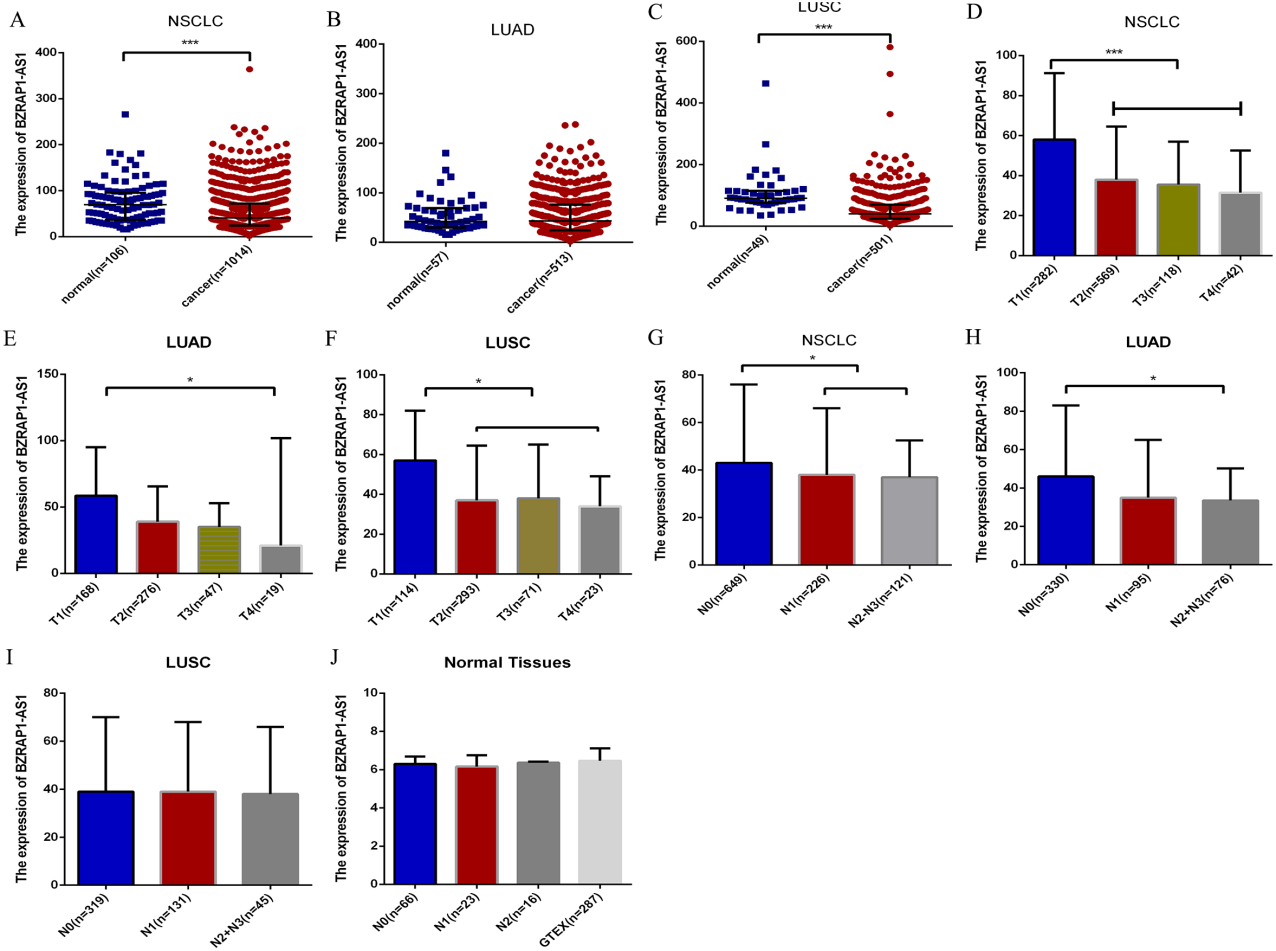
Bioinformatics analysis of the clinicopathological significance of *BZRAP1-AS1* in TCGA and GTEX database. The clinicopathological significance of *BZRAP1-AS1* in TCGA database. (A–C) *BZRAP1-AS1* expression level in cancer and adjacent normal tissues of NSCLC, LUAD and LUSC from TCGA data. (D–F) *BZRAP1-AS1* expression level in stage T1–T4 cancer tissues of NSCLC, LUAD and LUSC from TCGA data. (G–I) *BZRAP1-AS1* expression level in stage N0-N3 cancer tissues of NSCLC, LUAD and LUSC from TCGA data. (J) *BZRAP1-AS1* expression level in stage N0-N2 normal tissues of NSCLC from TCGA and GTEX normal tissues. *Statistical significance *P* < 0.05, **Statistical significance *P* < 0.01.

To validate the clinicopathological value of *BZRAP1-AS1* in NSCLC, we analyzed TCGA data using the SPSS, and Graphpad Prism. The results were in accordance with our RT-qPCR data. *BZRAP1-AS1* was low expressed in NSCLC cancer tissue compared with normal tissue (41.00 (24.00–72.00) *vs* 69.00 (37.00–95.00), *P* < 0.001) (Fig. 2A). In different histologic types, *BZRAP1-AS1* expression also was lower in LUSC cancer tissues than normal tissues [40.00 (24.00–69.00) *vs* 91.00 (76.00–114.00), *P* < 0.001], but no difference in LUAD (Figs. 2B–2C). In NSCLC, *BZRAP1-AS1* was lower in T2–T4 than T1 patients (38.00 (22.00–63.00) *vs* 58.00 (32.75–91.25), *P* < 0.001), N1-N3 than N0 patients (38.00 (23.00–63.00) *vs* 43.00 (24.00–76.00), *P* = 0.018) (Fig. 2D and 2G). In subgroup analysis, *BZRAP1-AS1* was lower in T2–T4 than T1 patients (38.50 (21.00–62.00) *vs* 58.50(32.00–95.00), *P* < 0.001), N1-N3 than N0 patients (34.00 (22.00–57.00) *vs* 46.00 (27.00–83.00), *P* = 0.001) in LUAD (Figs. 2E–2F). *BZRAP1-AS1* was lower in T2–T4 than T1 patients (37.00 (22.00–63.00) *vs* 57.00 (33.75–82.00), *P* < 0.001), but no difference in N stages in LUSC (Figs. 2H–2I). The expression of *BZRAP1-AS1* in normal tissues was no difference in N stages (Fig. 2J).

### *BZRAP1-AS1* participated in the pathological progress of NSCLC

**Figure 3:**
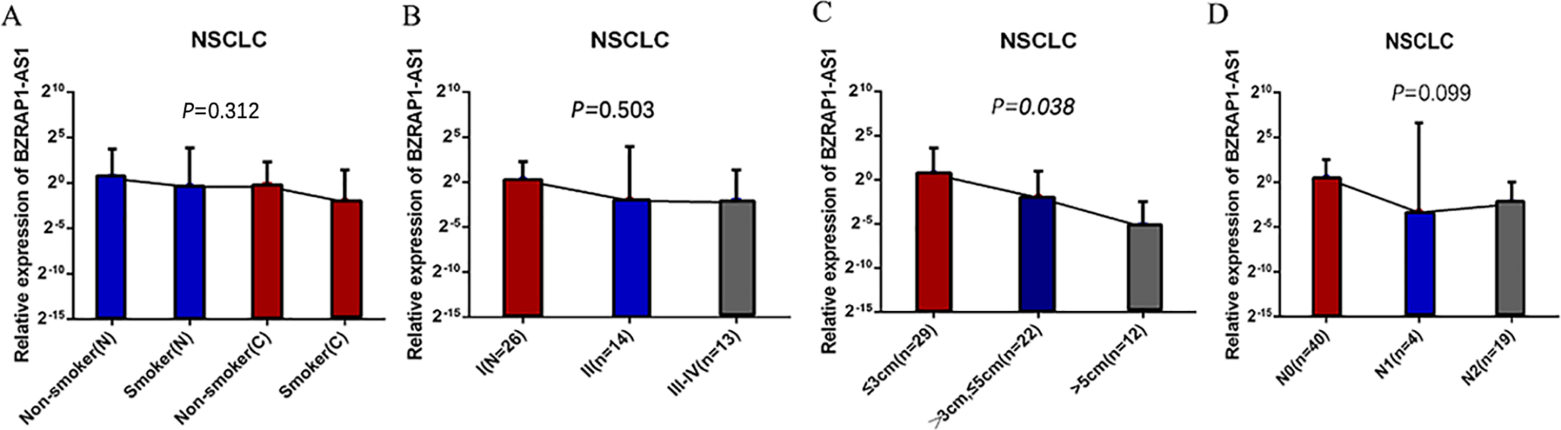
(A–D) The *BZRAP1-AS1* expression level stratified with smoking, TNM stage, tumor size, and N metastasis in 63 pairs NSCLC cancer and normal tissues detected by RT-qPCR.

Whereas *BZRAP1-AS1* was lower in tumor tissues than adjacent normal tissues in smokers, smoking was more meticulously analyzed. Compared with the adjacent normal tissues of nonsmokers, *BZRAP1-AS1* level reduced in tumor tissues of nonsmokers, lower in nonmalignant tissues of smokers, and the lowest in tumor tissues of smokers (Fig. 1H). In other words, *BZRAP1-AS1* levels decreased gradually with smoking from adjacent normal tissues to tumor tissues. Then, the relationship between TNM stage and *BZRAP1-AS1* in tumor tissues was more meticulously analyzed. *BZRAP1-AS1* levels decreased gradually from I to III–IV stage, from tumors less than three cm to tumors larger than five cm, and from N0 to N2 (Table 3 and Fig. 3).

**Table 3:** Combined analysis of *BZRAP1-AS1* expression and smoking history, tumor size and *N* stage.

**Variables**		**No. of cases**	***BZRAP1-AS1* expression (Median, 25–75 Percentile)**
Smoking	Tumor (T)/Adjacent Normal (N)		
NO	N	22	1.380, 0.165–13.416
NO	T	22	0.751, 0.086–4.913
YES	N	41	0.737, 0.061–14.324
YES	T	41	0.235, 0.025–2.695
TNM stage			
I		26	1.128, 0.081–4.847
II		14	0.237, 0.063–15.210
III–IV		23	0.001, 0.205–2.552
Tumor size(cm)			
≤3		29	1.528, 0.117–12.300
>3, ≤5		22	0.240, 0.078–1.973
>5		12	0.025, 0.001–0.182
N metastasis			
N0		40	1.354, 0.094–5.843
N1		4	0.098, 0.004–98.284
N2		19	0.187, 0.001–1.020

### Survival analysis

**Figure 4:**
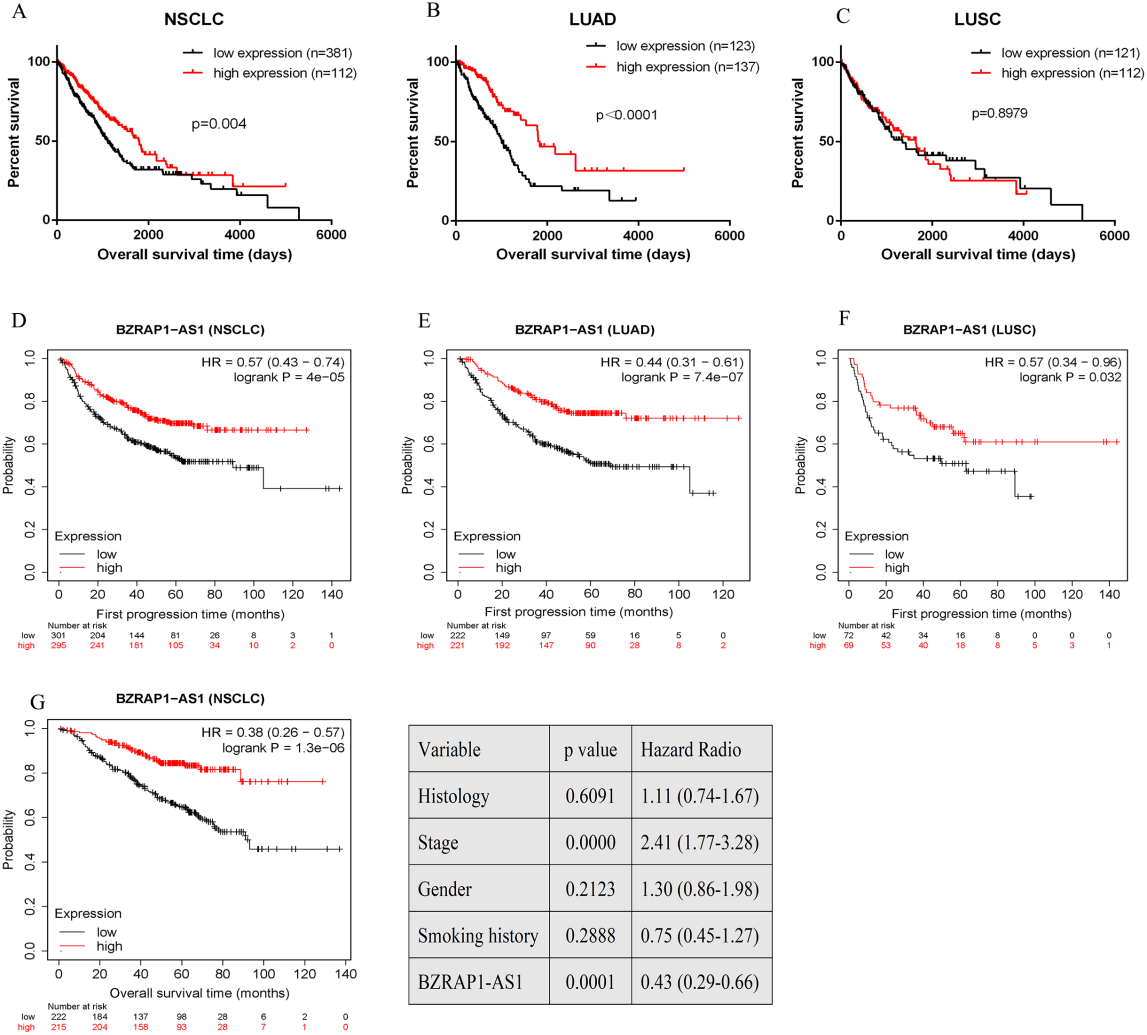
Bioinformatics analysis of the prognostic value of *BZRAP1-AS1* in TCGA database. (A–C) Kaplan–Meier survival analysis of overall survival of NSCLC based on TCGA cohort stratified by *BZRAP1-AS1* expression (bottom 25% *versus* top 25%). (D–F) Kaplan–Meier survival analysis of first progression of NSCLC based on TCGA cohort by Kaplan-Meier plotter online database. (G) Multivariate survival analysis including histology, TNM stage, gender, smoking history and *BZRAP1-AS1* expression (stratified by median) made by Kaplan-Meier plotter online database.

Overall survival (OS) and the first progression (FP) of NSCLC patients was analyzed by GraphPad Prism and Kaplan–Meier plotter in TCGA database. Quartile was selected as expression threshold to distinguish the high-expression and low-expression cohorts. Patients of low *BZRAP1-AS1* level shown poor prognosis. Further subgroup analysis, compared with the high-expression group, LUAD patients of low *BZRAP1-AS1* levels had shorter OS, but LUSC patients had no difference between two groups (Figs. 4A–4C). In KM Plotter, univariate analysis showed lower level *BZRAP1-AS1* was related to poor FP in both NSCLC, and in LUAD and LUSC subgroup patients (Figs. 4D–4F). Next, multivariate survival analysis results also indicated that *BZRAP1-AS1* was significantly associated with the prognosis of NSCLC patients (Fig. 4G).

### Overexpression of BZRAP1-AS1 suppressed proliferation, invasion, and migration of NSCLC cells

**Figure 5:**
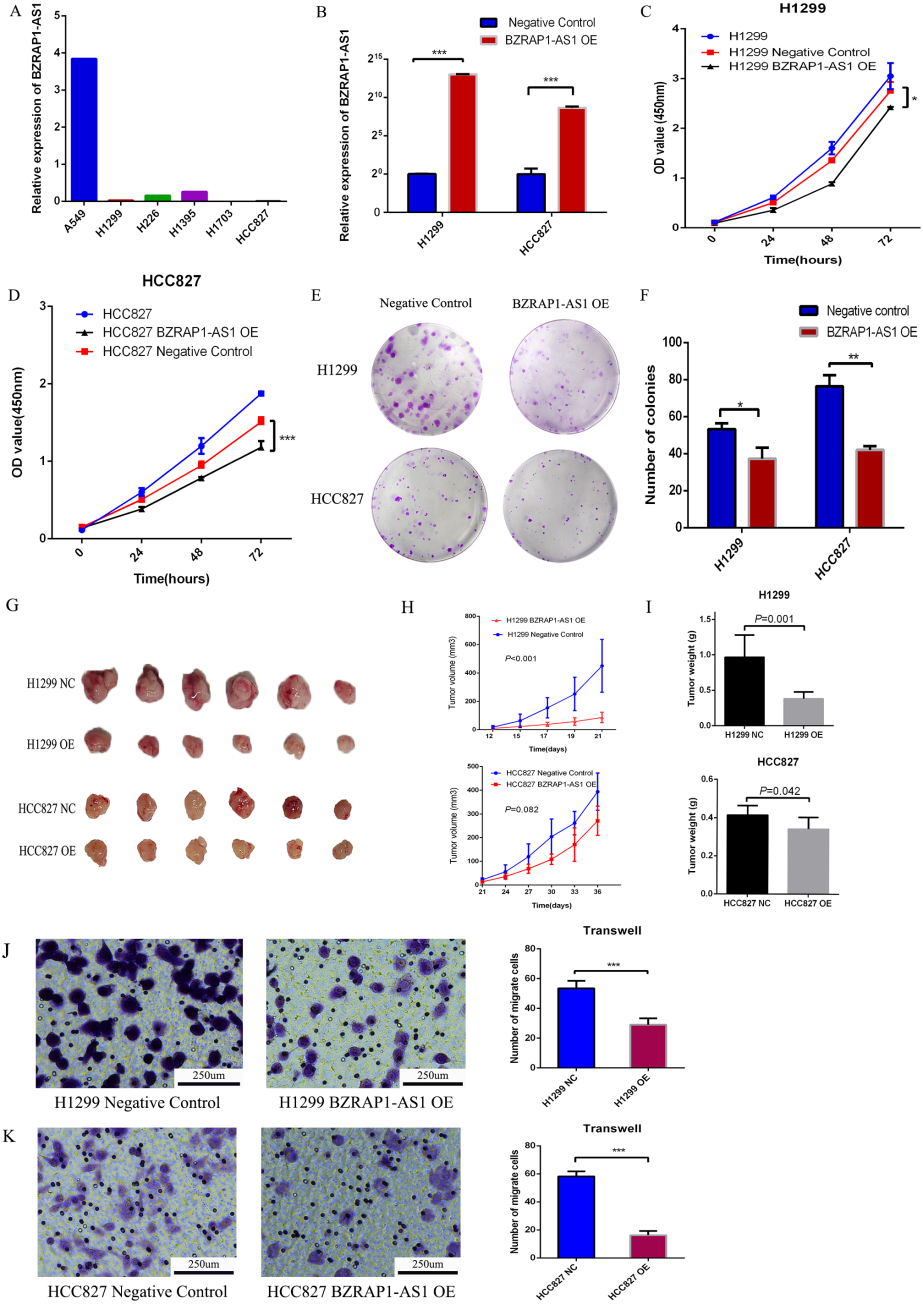
*BZRAP1-AS1* overexpressing suppresses H1299 and HCC827 proliferation and migration. (A) *BZRAP1-AS1* relative expression in NSCLC cell lines detected by RT-qPCR. (B) qPCR detection of *BZRAP1-AS1* overexpression in H1299 and HCC827. OE, overexpression; NC, negative control (C–D) CCK-8 assays to measure the proliferation abilities of *BZRAP1-AS1* overexpressing H1299 and HCC827 cells. (E-F) Colony formation ability of *BZRAP1-AS1* overexpressing H1299 and HCC827 cells. (G–H) Mice Xenograft tumor growth of *BZRAP1-AS1* overexpressing H1299 and HCC827 cells in BALB/c-nu mice, *n* = 6. (I) The tumor weight from *BZRAP1-AS1* overexpressing and control cells. (J–K) Transwell assay of *BZRAP1-AS1* overexpressing H1299 and HCC827 cells. (×10). *Statistical significance *P* < 0.05, **Statistical significance *P* < 0.01, ***Statistical significance *P* < 0.001.

To validate the role of *BZRAP1-AS1* in NSCLC, we firstly detected the level of *BZRAP1-AS1* in NSCLC cell lines (A549, H1299, H226, H1395, H1703, HCC827) by RT-qPCR. Except for A549 cells, the expression of *BZRAP1-AS1* was very low in NSCLC cells (Fig. 5A). Second, H1299 and HCC827 cells with low *BZRAP1-AS1* levels were selected to overexpress *BZRAP1-AS1*. As the results of RT-qPCR, the relative expression of *BZRAP1-AS1* in H1299 OE was more than 8000 times higher than H1299 NC, and HCC827 OE nearly 400 times higher than HCC827 NC (Fig. 5B). The results of cell growth assay showed BZRAP1-AS1 over-expressing cells were slower than control cells (Figs. 5C–5D). The results of colony formation assay showed colony number of H1299 OE and HCC827 OE were less than respective controls (37.33 ± 3.38 *vs* 53.33 ± 1.76, *P* = 0.014), less than HCC827 NC (42.00 ± 1.16 *vs* 76.33 ± 3.48, *P* < 0.001) (Figs. 5E–5F). These results indicated that overexpression of *BZRAP1-AS1* inhibited proliferation of H1299 and HCC827 cells in *vitro*. In *vivo*, the results of mice tumor growth curve showed H1299 OE were slower than H1299 NC groups (*P* < 0.001), and HCC827 OE than HCC827 NC group (*P* = 0.082) (Figs. 5G–5H). The tumor weight of H1299 OE was less than H1299 NC (0.382 ± 0.039 *vs* 0.970 ± 0.127, *P* = 0.001), and HCC827 OE less than HCC827 NC group (0.342 ± 0.025 *vs* 0.415 ± 0.019, *P* = 0.042) (Fig. 5I). Additionally, the migration ability of H1299 OE cells was weaker than H1299 NC group (29.000 ± 2.160 *vs* 53.500 ± 2.533, *P* < 0.001), similar to HCC827 OE cells compared with HCC827 NC cells (16.250 ± 1.493 *vs* 58.250 ± 1.797, *P* < 0.001). Thus, overexpression of BZRAP1-AS1 inhibited migration of H1299 and HCC827 cells (Figs. 5J–5K).

### Transcriptomic analysis of mice xenograft tumor tissues and co-expression analysis

**Figure 6:**
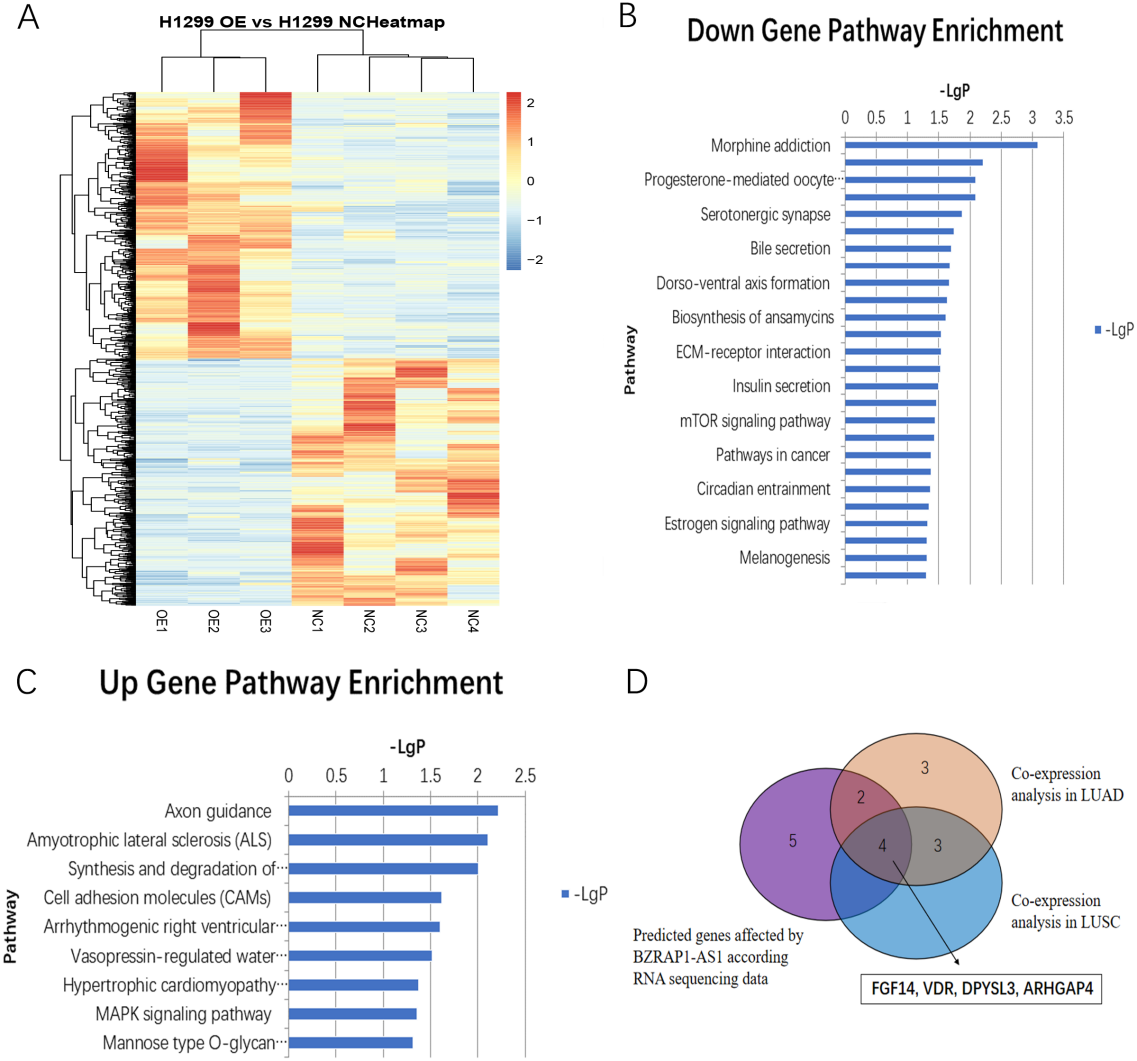
(A) Heatmap of differentially expressed genes between H1299 NC and H1299 OE group in RNAseq of mice Xenograft tumor tissues (405 up-regulated genes and 376 down-regulated genes). (B–C) Statistics of KEGG pathway enrichment analysis results for up-regulated and down-regulated genes with overexpression of *BZRAP1-AS1*. (D) Venn diagram for twelve predicted genes affected by *BZRAP1-AS1* in RNA sequencing data and co-expression analysis in LUAD and LUSC.

RNA-seq analysis of mice tumor tissues derived from BZRAP1-AS1 over-expressing H1299 cells and the control cells showed there were 405 up-regulated genes and 376 down-regulated genes (Fig. 6A). And GO analysis showed BZRAP1-AS1 correlated with metal ion binding, translation factor activity (RNA binding), translation repressor activity (nucleic acid binding), coreceptor activity involved in Wnt signaling pathway (Figs. S1A–S1B). The results of KEGG pathway enrichment analysis showed *BZRAP1-AS1* correlated with tumor-associated signaling pathways including cell adhesion molecules (CAM), MAPK signaling pathway, mTOR signaling pathway, pathways in cancer (Figs. 6B–6C). According to the above results, we traced to the KEGG Pathway/GO annotation of differentially expressed genes, further predicted the genes affected by *BZRAP1-AS1*. Finally, twelve genes were predicted, including five down-regulated genes RPS6KA6, LUM, TWIST1, PRKACB, TWIST2, and seven up-regulated genes PPM1A, FGF14, CNTN1, L1CAM, VDR, DPYSL3, ARHGAP4 (Table S2). Then co-expression analysis was performed to validate the correlation between BZRAP1-AS1 and twelve predicted genes in LUAD and LUSC preliminarily, and results showed *BZRAP1-AS1* was positively correlated with RPS6KA6, LUM, PRKACB, FGF14, VDR, DPYSL3 and ARHGAP4 (Figs. S2–S3). In summary, FGF14, VDR, DPYSL3 and ARHGAP4 may be the genes affected by *BZRAP1-AS1* to function in LUAD and LUSC (Fig. 6D).

## Discussion

Increasing studies show lncRNA is closely correlated with diagnosis, prognosis, and drug resistance of tumors (Goodall & Wickramasinghe, 2021; Zhang et al., 2020; Sun et al., 2020). LncRNA may be a next breakthrough in the fight against tumors (Tan et al., 2021b; Bhan, Soleimani & Mandal, 2017). *BZRAP1-AS1* is a lncRNA being found in recent years. There are few studies on the relationship between *BZRAP1-AS1* and tumors. In hepatocellular carcinoma (HCC), *BZRAP1-AS1* is high expression and knockdown of *BZRAP1-AS1* inhibits cell proliferation, migration, and angiogenesis in HUVEC (Wang et al., 2019b), which suggests *BZRAP1-AS1* may play a role in promoting tumorigenesis. But in cervical cancer and pancreatic cancer, low level of *BZRAP1-AS1* is correlated with poor OS (Giulietti et al., 2018; Zheng et al., 2020b), which suggests that *BZRAP1-AS1* may play a role in inhibiting tumorigenesis. These inconsistent results indicate that *BZRAP1-AS1* exerts different functions in the different tumors.

For lung cancer, the relationship between *BZRAP1-AS1* and prognosis is only studied by bioinformatics analysis in lung adenocarcinoma, and functional studies of *BZRAP1-AS1* in lung cancer are very limited (Wang et al., 2019a; Tang et al., 2021). NSCLC patients account for 85% of lung cancer patients. Therefore, we focused on the relationship between *BZRAP1-AS1* and NSCLC. Firstly, we detected the level of *BZRAP1-AS1* in tumor tissues and adjacent normal tissues of 63 NSCLC patients to investigate whether there is expression difference between them. We find *BZRAP1-AS1* level is lower in tumor tissues than adjacent normal tissues. Further subgroup analysis exhibits that *BZRAP1-AS1* is lower in tumor tissues than adjacent normal tissues in smoking group and not different in nonsmoking group, which suggests smoking may cause a decrease of *BZRAP1-AS1* in tumor tissues. Then smoking is more meticulously divided and the effects of smoking on *BZRAP1-AS1* expression are analyzed in tumor tissues and adjacent normal tissues. Compared with adjacent normal tissues of nonsmokers, *BZRAP1-AS1* level reduced in tumor tissues of nonsmokers, lower in nonmalignant tissues of smokers, and the lowest in tumor tissues of smokers. Thus, we speculate BZRAP1-AS1 has a greater impact on in the process of smoking-related lung cancer. In addition, subgroup analysis shows *BZRAP1-AS1* is lower in tumor tissues than adjacent normal tissues in male and LUSC groups and not different in female and LUAD groups. We think these differences of *BZRAP1-AS1* in the male and LUSC groups have nothing to do with gender and pathological types, but because there is a higher proportion of smokers in male than female patients and in LUSC than LUAD groups (87.0% *vs* 5.9%, 56.5% *vs* 11.8%). Also, smoking is a major high-risk factor for LUSC (Pesch et al., 2012). Then subgroup analysis in tumor tissues shows *BZRAP1-AS1* is lower in T2–T4 than in T1, and in N1-N2 than in N0. In other words, *BZRAP1-AS1* level is high in small tumors and low in big tumors, and high in tumors without N2 lymph nodes metastasis and low in tumors with N2 lymph nodes metastasis. When TNM stage, tumor size, and N metastasis more meticulously analyzed, *BZRAP1-AS1* expression reduces gradually from I to III-IV, from ≤ three cm to >5 cm, from N0 to N2. In other words, the levels of *BZRAP1-AS1* associated with the malignancy of NSCLC. So, we deduce that *BZRAP1-AS1* participates in the pathological progress of NSCLC. To validate the hypothesis, TCGA data is analyzed. The results are consistent with our data. In addition, the survival analysis results show *BZRAP1-AS1* is related to OS in LUAD, and FP in LUAD and LUSC. Patients with high level of *BZRAP1-AS1* have a better prognosis, which is consistent with the literatures (Giulietti et al., 2018; Zheng et al., 2020b).

In addition, we study the expression of *BZRAP1-AS1* in NSCLC cell lines. In six NSCLC cell lines, Except for A549 cells, the expression of BZRAP1-AS1 is very low in five cells and only high in A549 cells. This result is consistent with that of tumor tissues. That is to say that the expression of BZRAP1-AS1 is very low in tumors. As for A549 cells, we think it is just a one example. How does *BZRAP1-AS1* affect pathological process of NSCLC? We overexpress *BZRAP1-AS1* in NSCLC cells to study the effect of *BZRAP1-AS1* on cell proliferation, invasion, and migration in *vitro* and in *vivo*. The data indicate that overexpressing *BZRAP1-AS1* indeed suppresses proliferation, invasion, and metastasis of NSCLC cells. This further confirms *BZRAP1-AS1* plays an anticancer role by inhibiting proliferation, invasion, and migration. This is consistent with the data in cervical cancer and pancreatic cancer (Zheng et al., 2020b; Tang et al., 2021).

To further explore which genes are affected by *BZRAP1-AS1* in NSCLC, transcriptome analysis was performed. By GO enrichment analysis and KEGG pathway enrichment analysis, we screened twelve potentially relevant genes: RPS6KA6, LUM, TWIST1, PRKACB, TWIST2, PPM1A, FGF14, CNTN1, L1CAM, VDR, DPYSL3, ARHGAP4. After co-expression verification, four genes were finally concerned, namely FGF14, VDR, DPYSL3 and ARHGAP4. According to available literatures, these four genes play a role in suppressing tumor (Babina & Turner, 2017; Turner & Grose, 2010; Turkowski et al., 2020; Campbell & Trump, 2017; Yang et al., 2018; Shen et al., 2019). So we guess *BZRAP1-AS1* exerts its tumor suppressive function by influencing above four genes. *BZRAP1-AS1* is expected to become a new therapeutic target for NSCLC.

## Conclusion

*BZRAP1-AS1* expression negatively correlates with malignancy grades of NSCLC. BZRAP1-AS1 plays an anticancer role by inhibiting cell proliferation, invasion, and metastasis, and has promising prognostic value in NSCLC. BZRAP1-AS1 may serve as a potentially diagnostic marker and therapeutic target for NSCLC.

##  Supplemental Information

10.7717/peerj.13871/supp-1 Figure S1 Statistics of GO molecular function (MF) enrichment results for up-regulated and down-regulated genes with overexpression of *BZRAP1-AS1*

10.7717/peerj.13871/supp-2 Figure S2 Co-expression analysis in LUAD for *BZRAP1-AS1* and twelve predicted target genes RPS6KA6, LUM, TWIST1, PRKACB, TWIST2, PPM1A, FGF14, CNTN1, L1CAM, VDR, DPYSL3, ARHGAP4

10.7717/peerj.13871/supp-3 Figure S3 Co-expression analysis in LUSC for *BZRAP1-AS1* and twelve predicted target genes RPS6KA6, LUM, TWIST1, PRKACB, TWIST2, PPM1A, FGF14, CNTN1, L1CAM, VDR, DPYSL3, ARHGAP4

10.7717/peerj.13871/supp-4 Table S1 The proportion of smoking patients in sex and histologic type

10.7717/peerj.13871/supp-5 Table S2 Annotation information and expression changes of predicted target genes with the overexpression of *BZRAP1-AS1* (Mouse tumor tissue sequencing data)

10.7717/peerj.13871/supp-6 Supplemental Information 1 ARRIVE 2.0 checklist

10.7717/peerj.13871/supp-7 Supplemental Information 2 Raw RNAseq data from mice tumor Eexpression differential analysis for RNAseq of mice tumor.

10.7717/peerj.13871/supp-8 Data S1 Raw data

## Additional Information and Declarations

### Data Availability

The following information was supplied regarding data availability: The raw data are available in the Supplemental Files.
