# Supplementary figures and images for: Long non-coding RNA BZRAP1-AS1 functions in malignancy and prognosis for non-small-cell lung cancer
Source: PeerJ. 2022 Aug 23;10:e13871. doi: 10.7717/peerj.13871 (PMC9415519; doi:10.7717/peerj.13871)

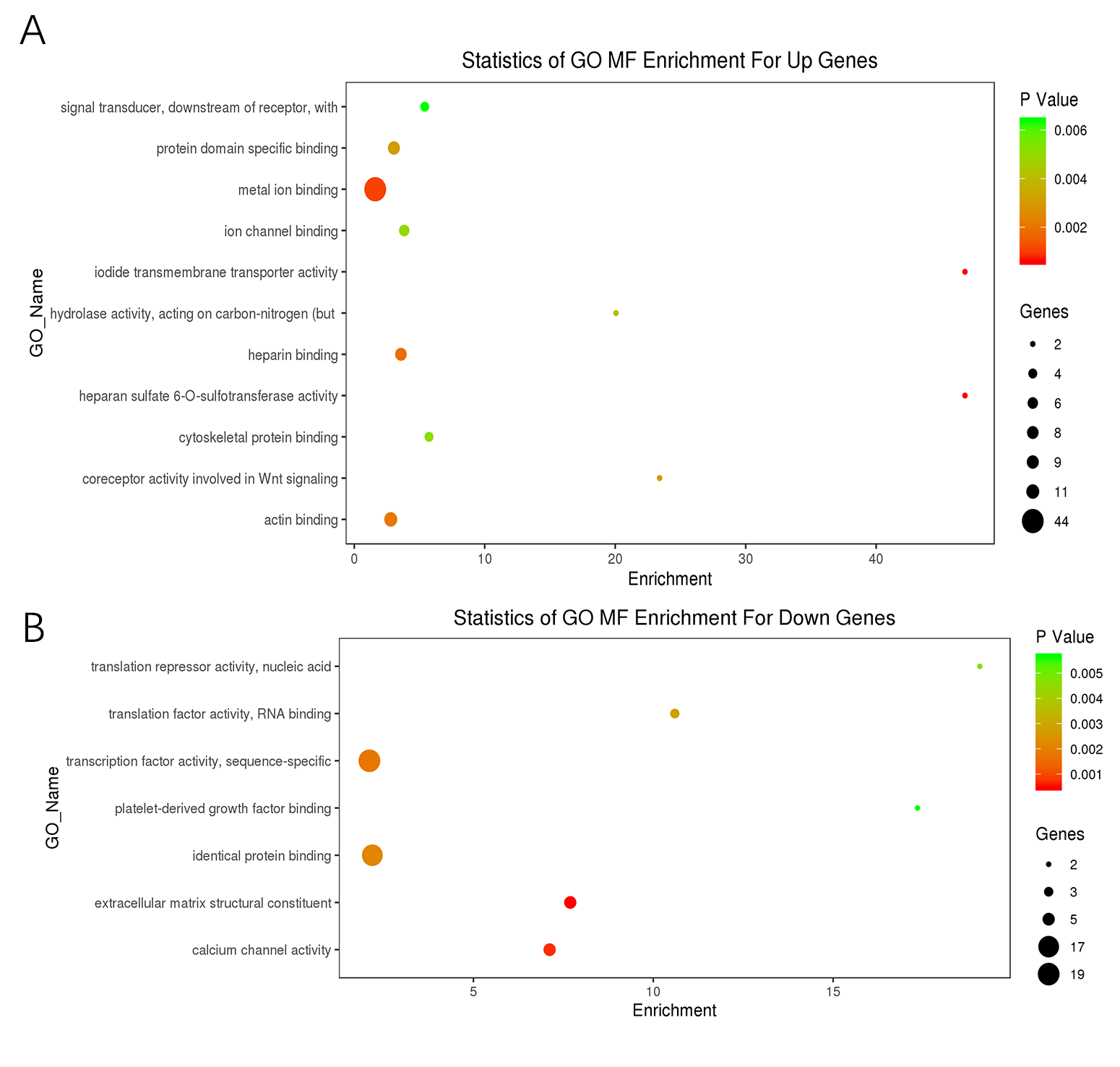

Supplement: Figure S1 [file peerj-10-13871-s001.png]

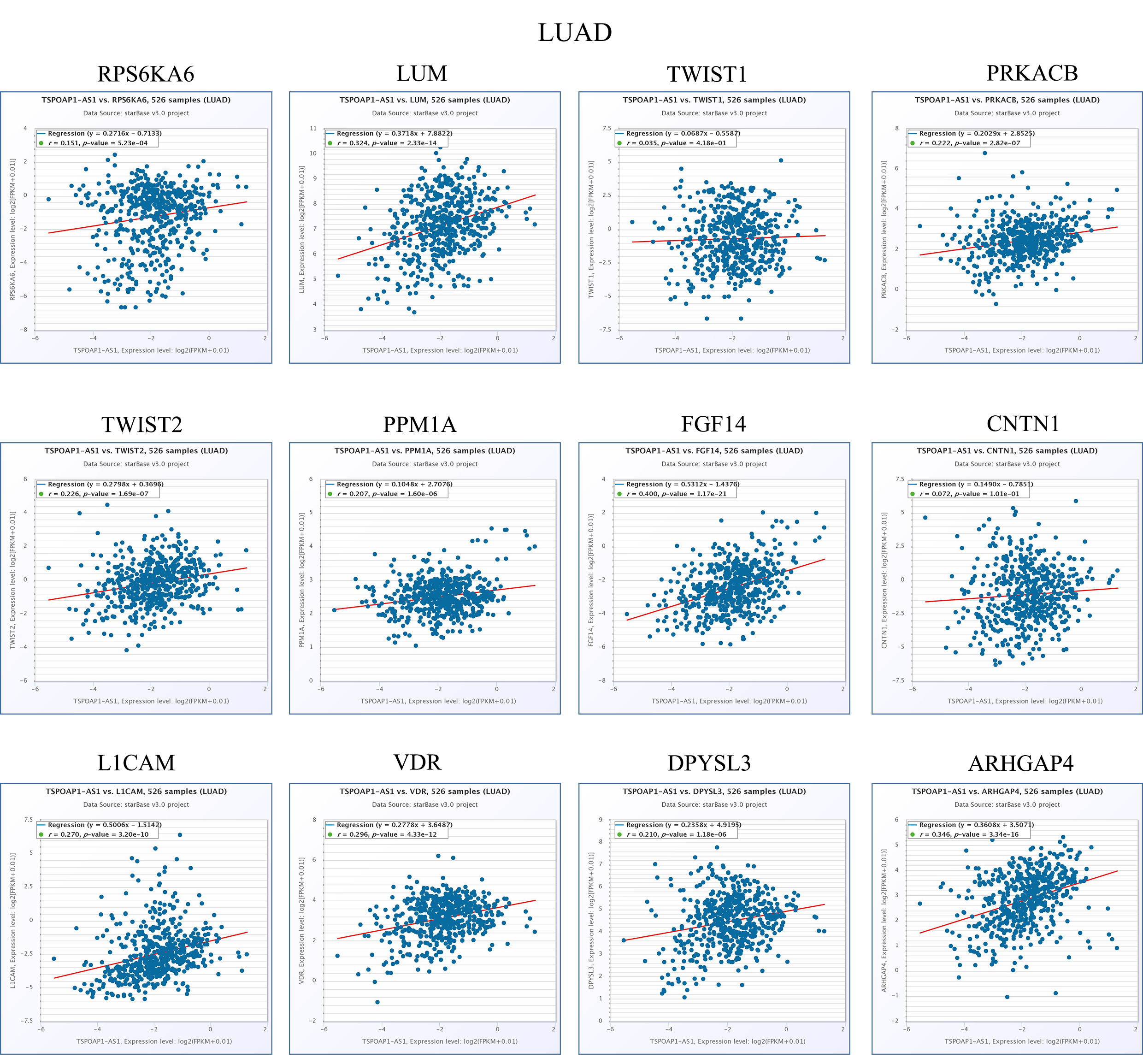

Supplement: Figure S2 [file peerj-10-13871-s002.png]

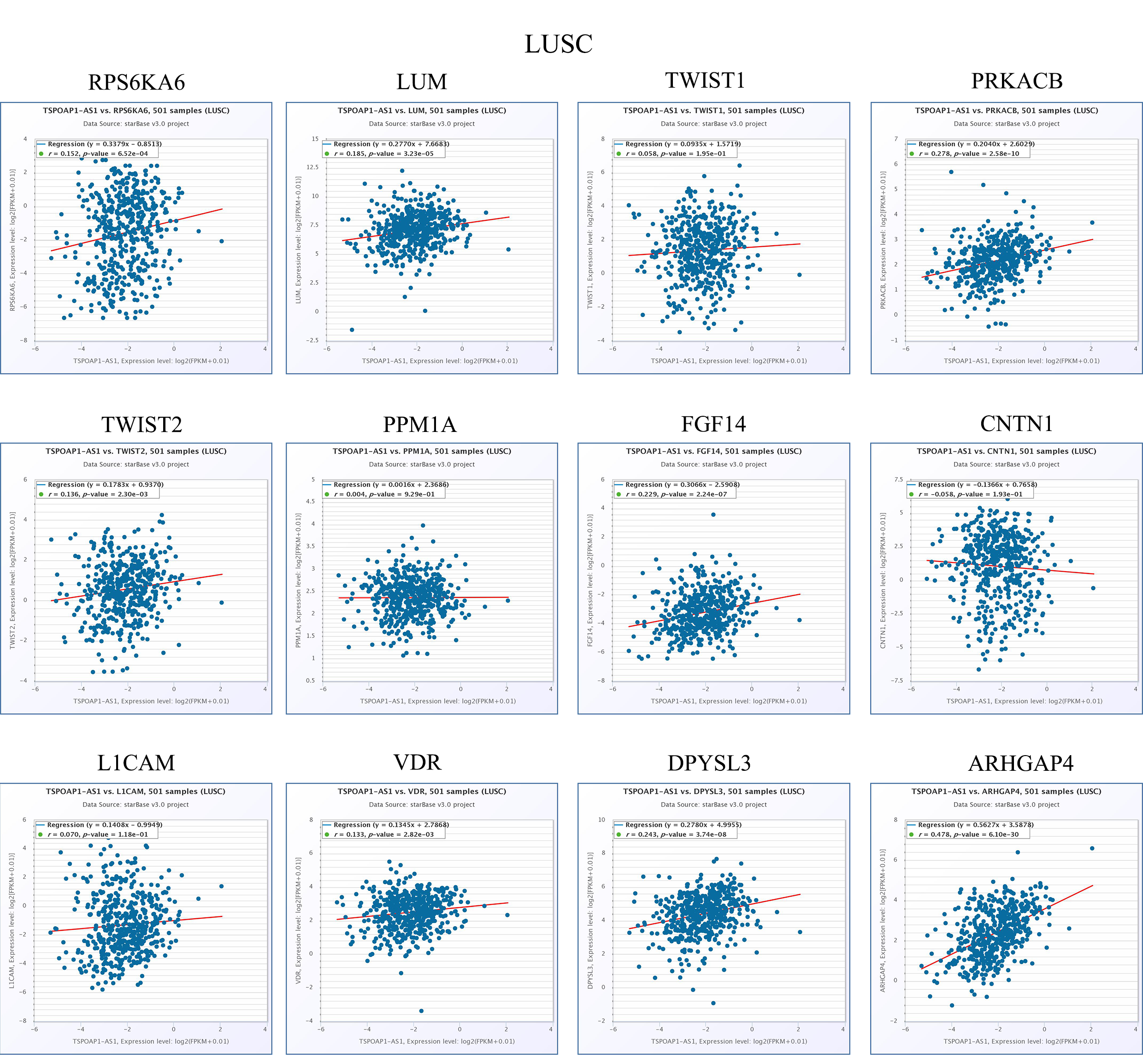

Supplement: Figure S3 [file peerj-10-13871-s003.png]

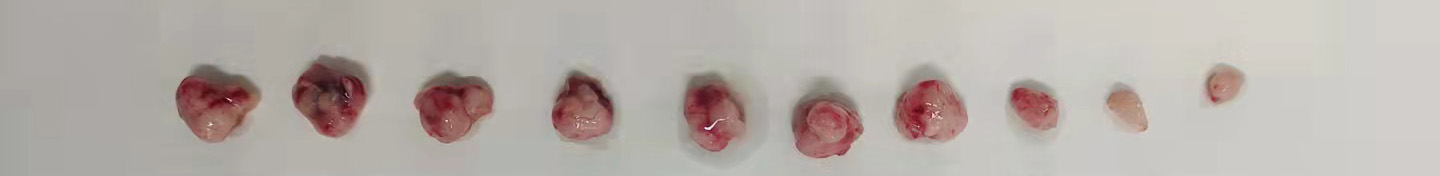

Supplement: Data S1 [file peerj-10-13871-s008.zip › source data/Tumor growth in nude mice/H1299/1299 Negative Control .jpg]

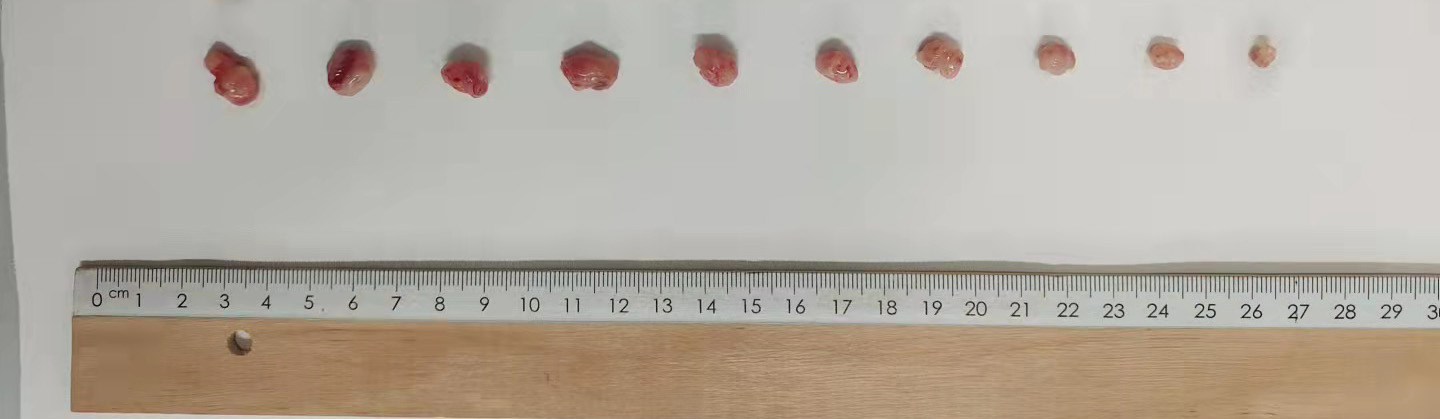

Supplement: Data S1 [file peerj-10-13871-s008.zip › source data/Tumor growth in nude mice/H1299/H1299 BZRAP1-AS1 OE.jpg]

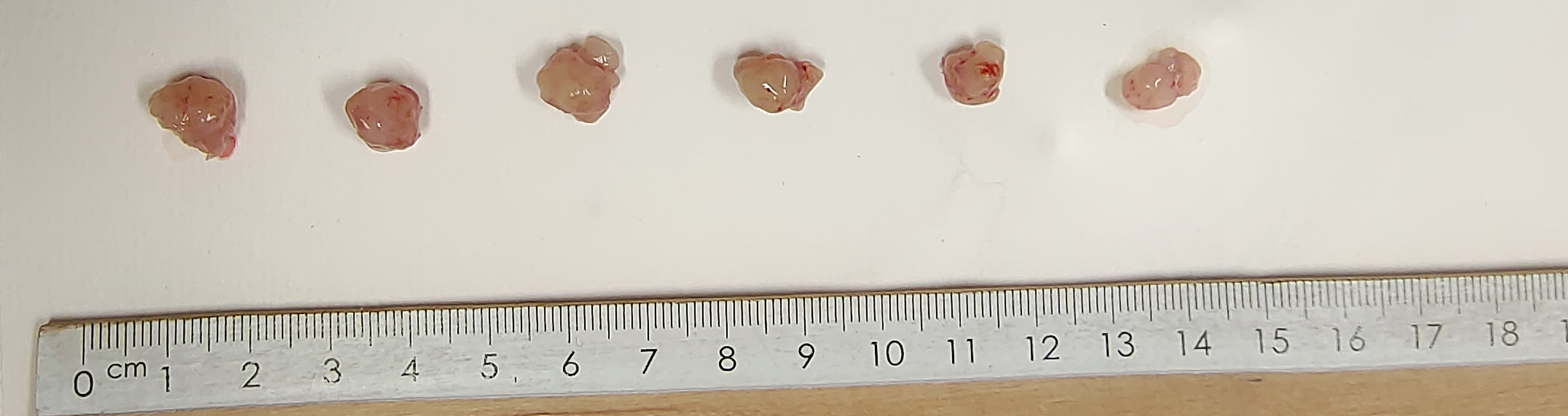

Supplement: Data S1 [file peerj-10-13871-s008.zip › source data/Tumor growth in nude mice/HCC827/HCC827-BZRAP1-AS1 OE.jpg]

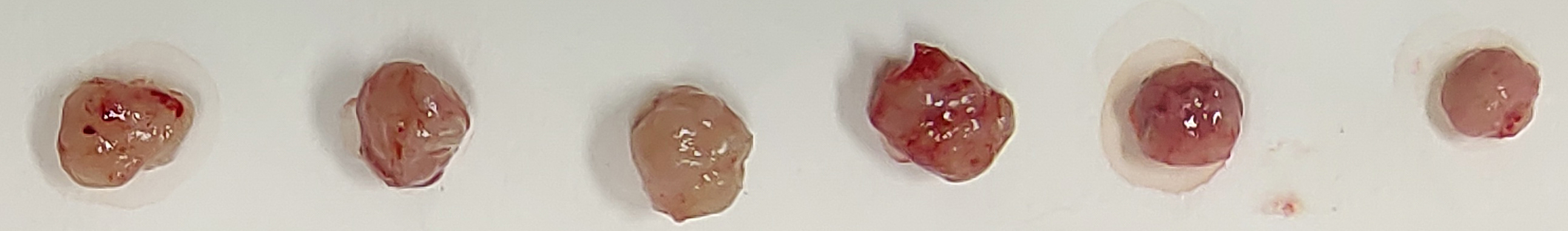

Supplement: Data S1 [file peerj-10-13871-s008.zip › source data/Tumor growth in nude mice/HCC827/HCC827-Negative Control.jpg]

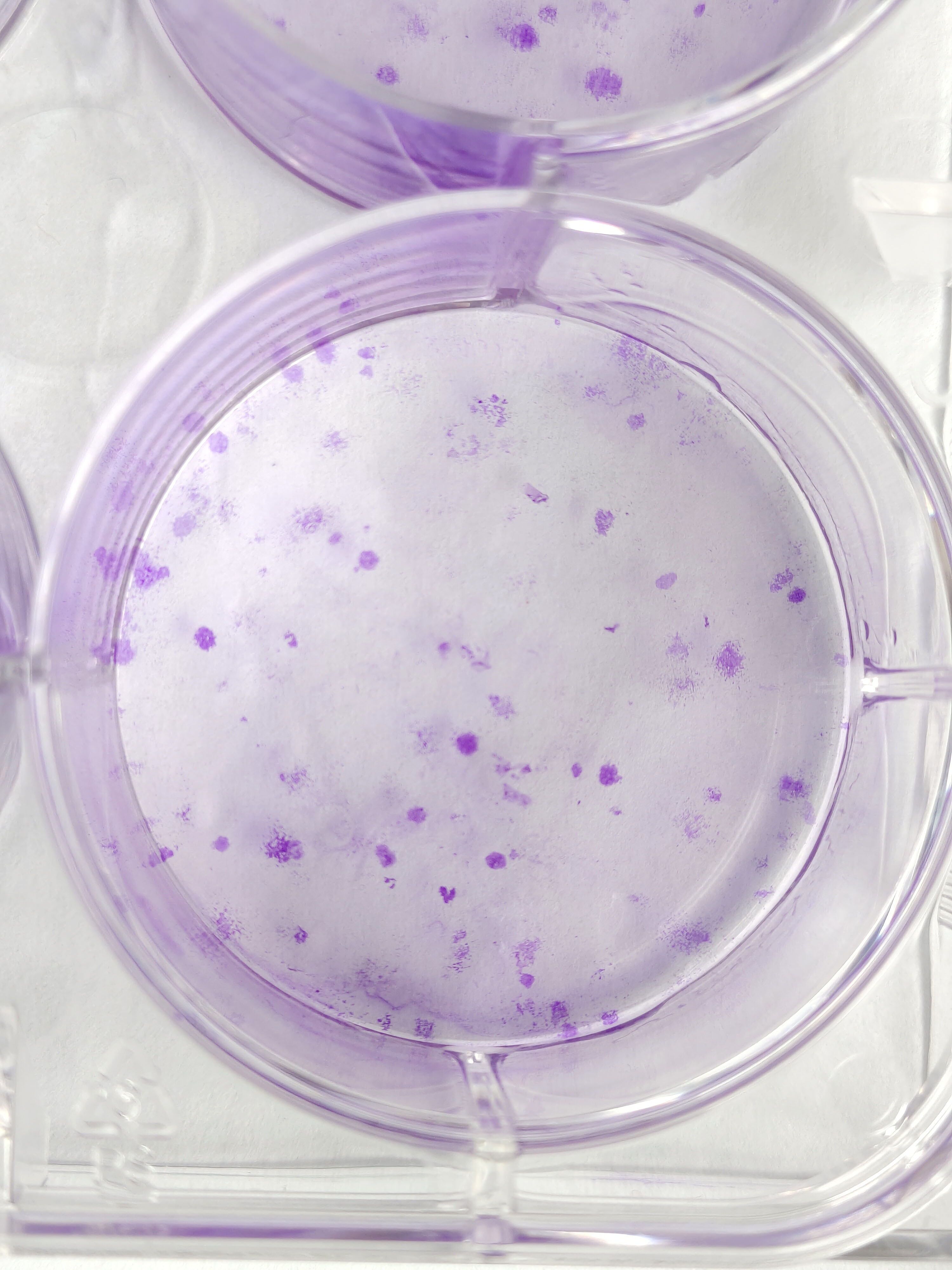

Supplement: Data S1 [file peerj-10-13871-s008.zip › source data/cell assay/Colony formation assay/H1299/H1299 BZRAP1-AS1 OE/1.jpg]

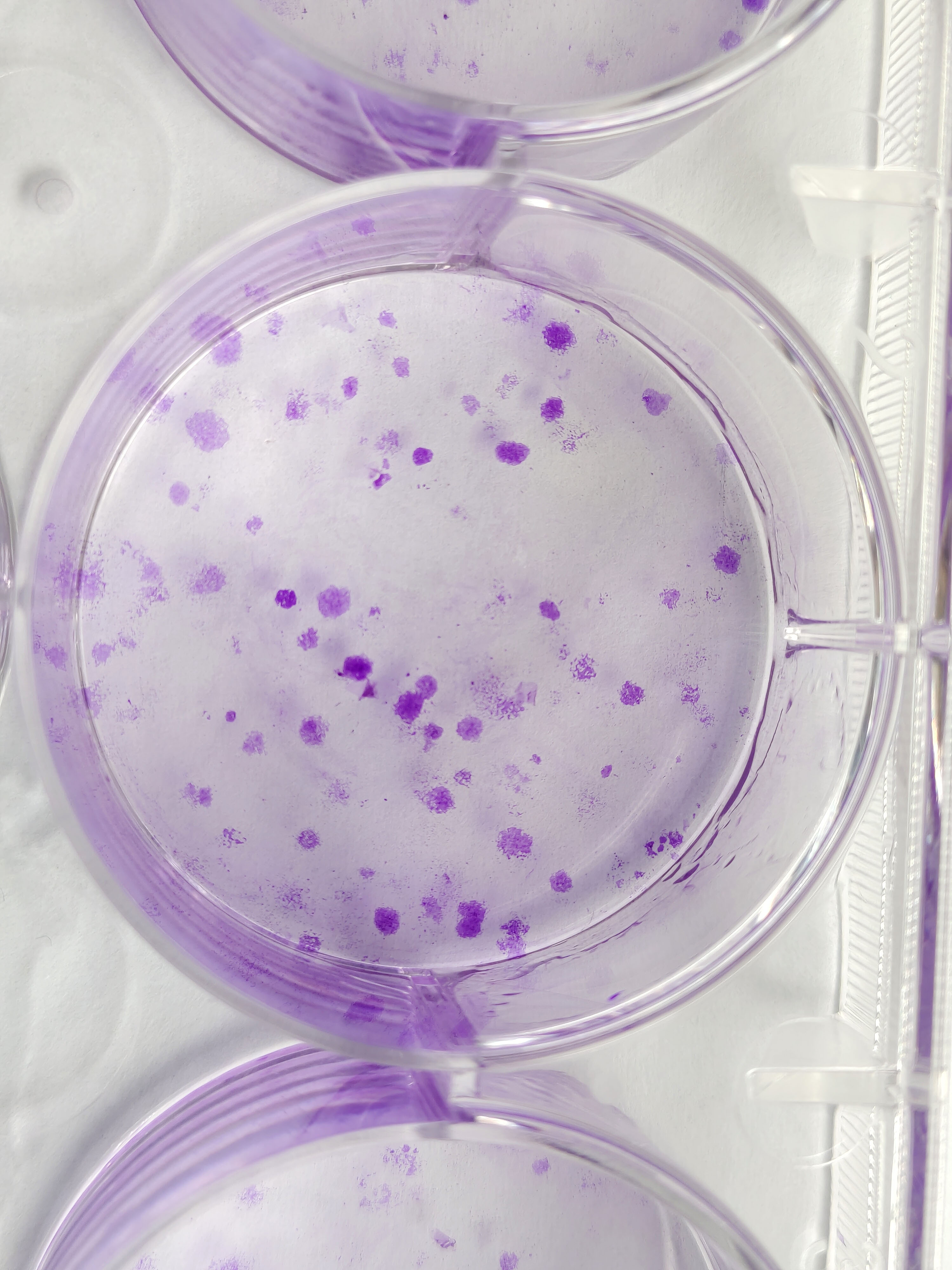

Supplement: Data S1 [file peerj-10-13871-s008.zip › source data/cell assay/Colony formation assay/H1299/H1299 BZRAP1-AS1 OE/2.jpg]

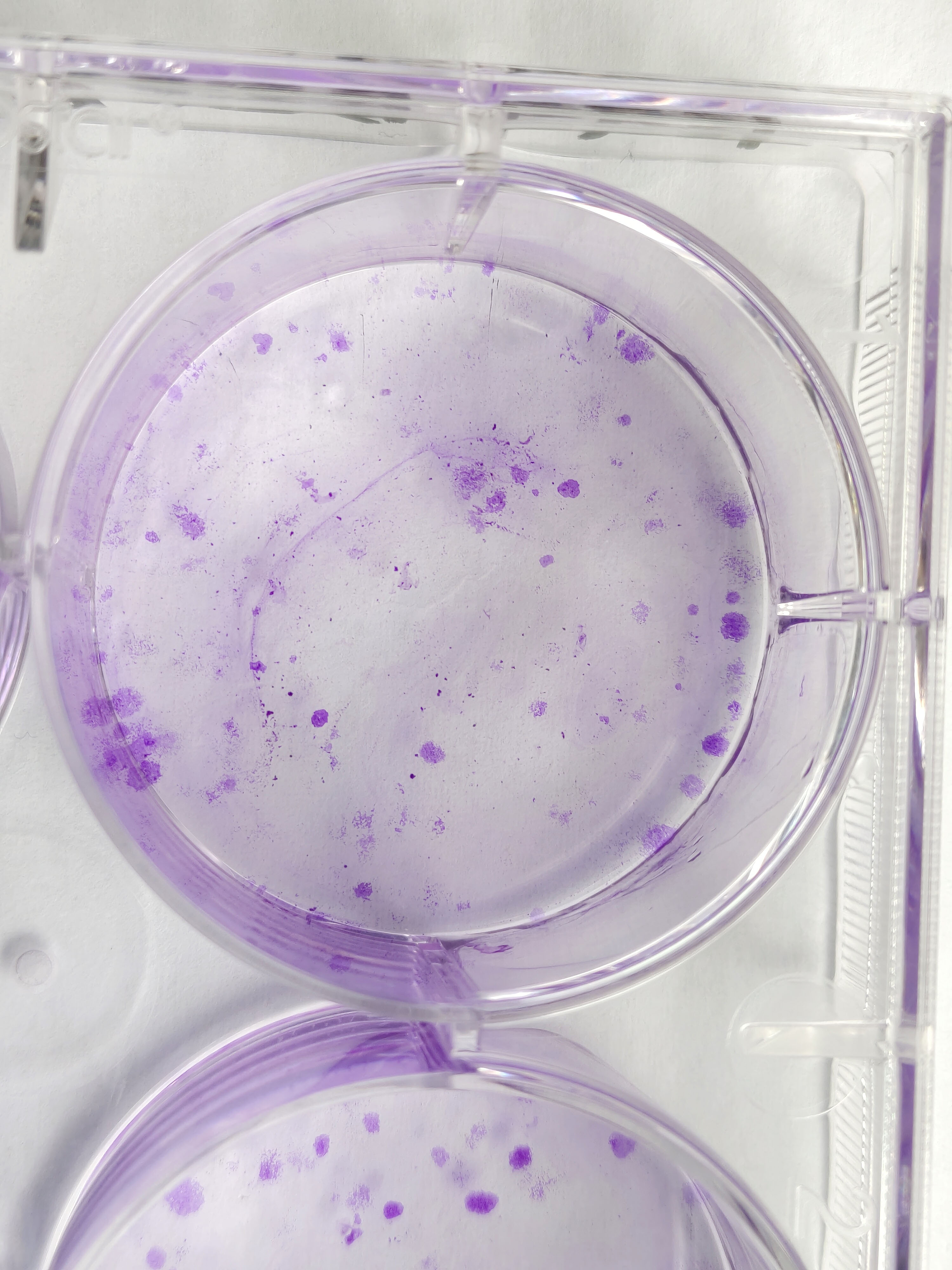

Supplement: Data S1 [file peerj-10-13871-s008.zip › source data/cell assay/Colony formation assay/H1299/H1299 BZRAP1-AS1 OE/3.jpg]

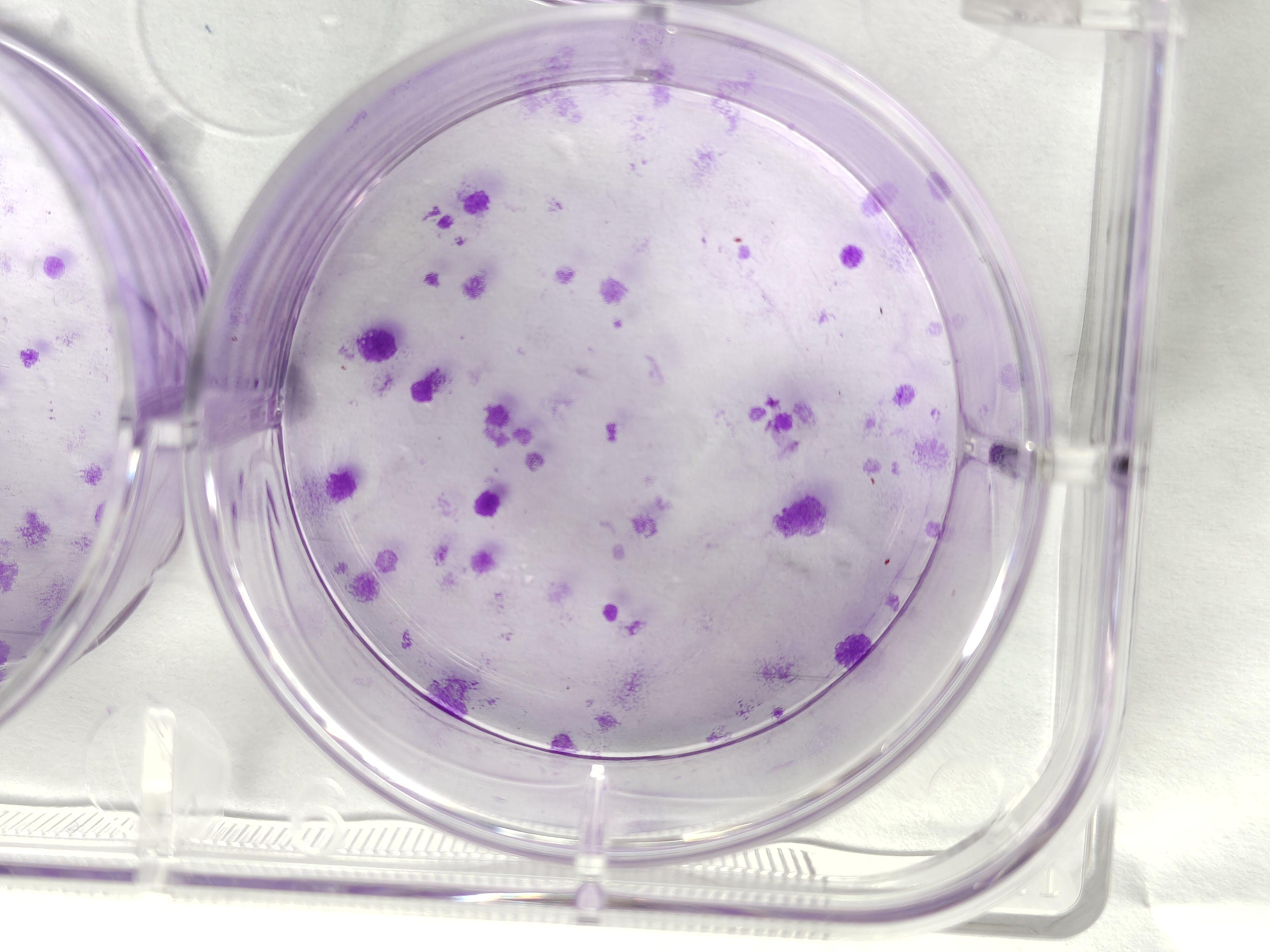

Supplement: Data S1 [file peerj-10-13871-s008.zip › source data/cell assay/Colony formation assay/H1299/H1299 NC/1299 bz nc1 6.26.jpg]

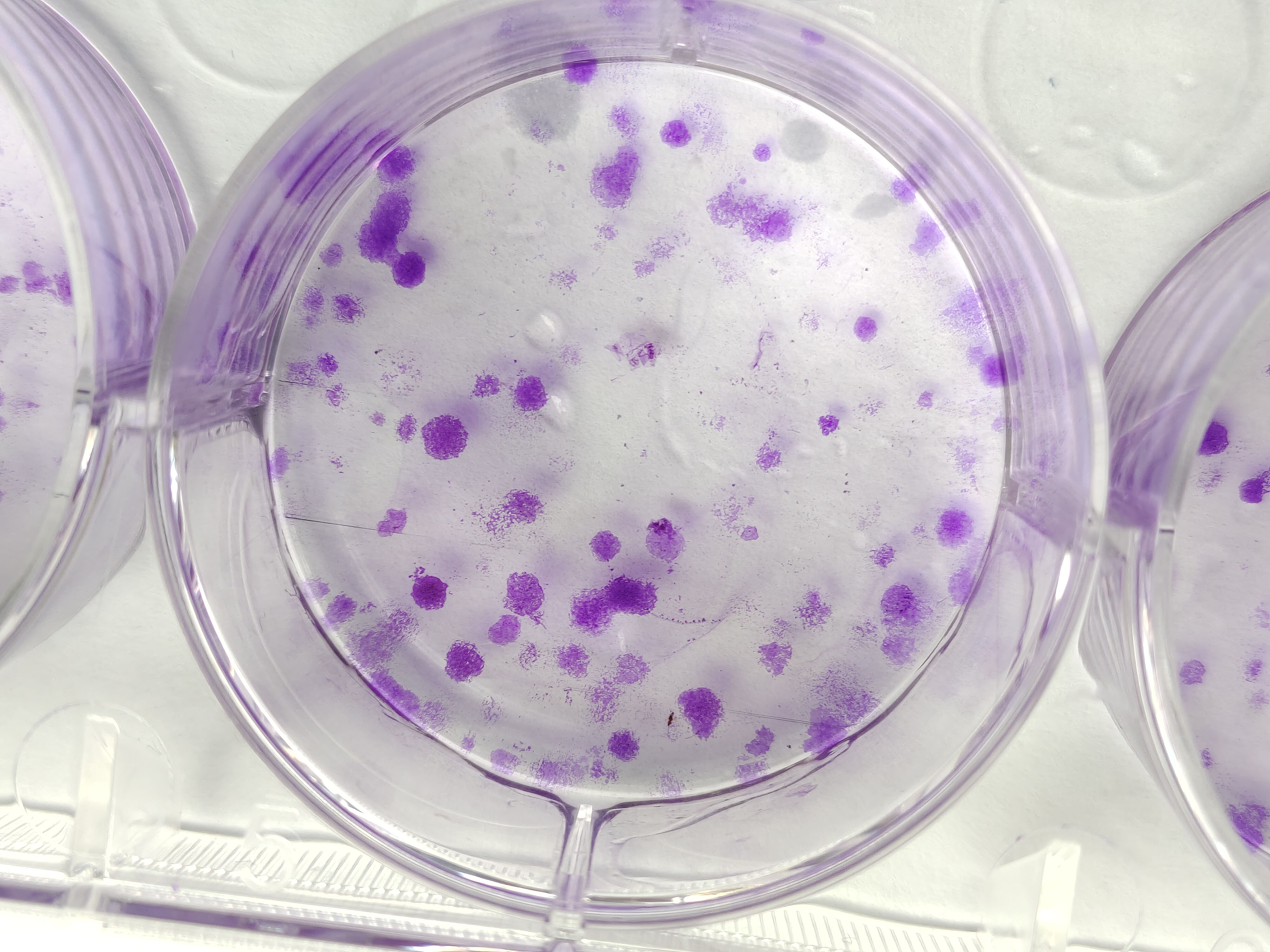

Supplement: Data S1 [file peerj-10-13871-s008.zip › source data/cell assay/Colony formation assay/H1299/H1299 NC/1299 bz nc2 6.26.jpg]

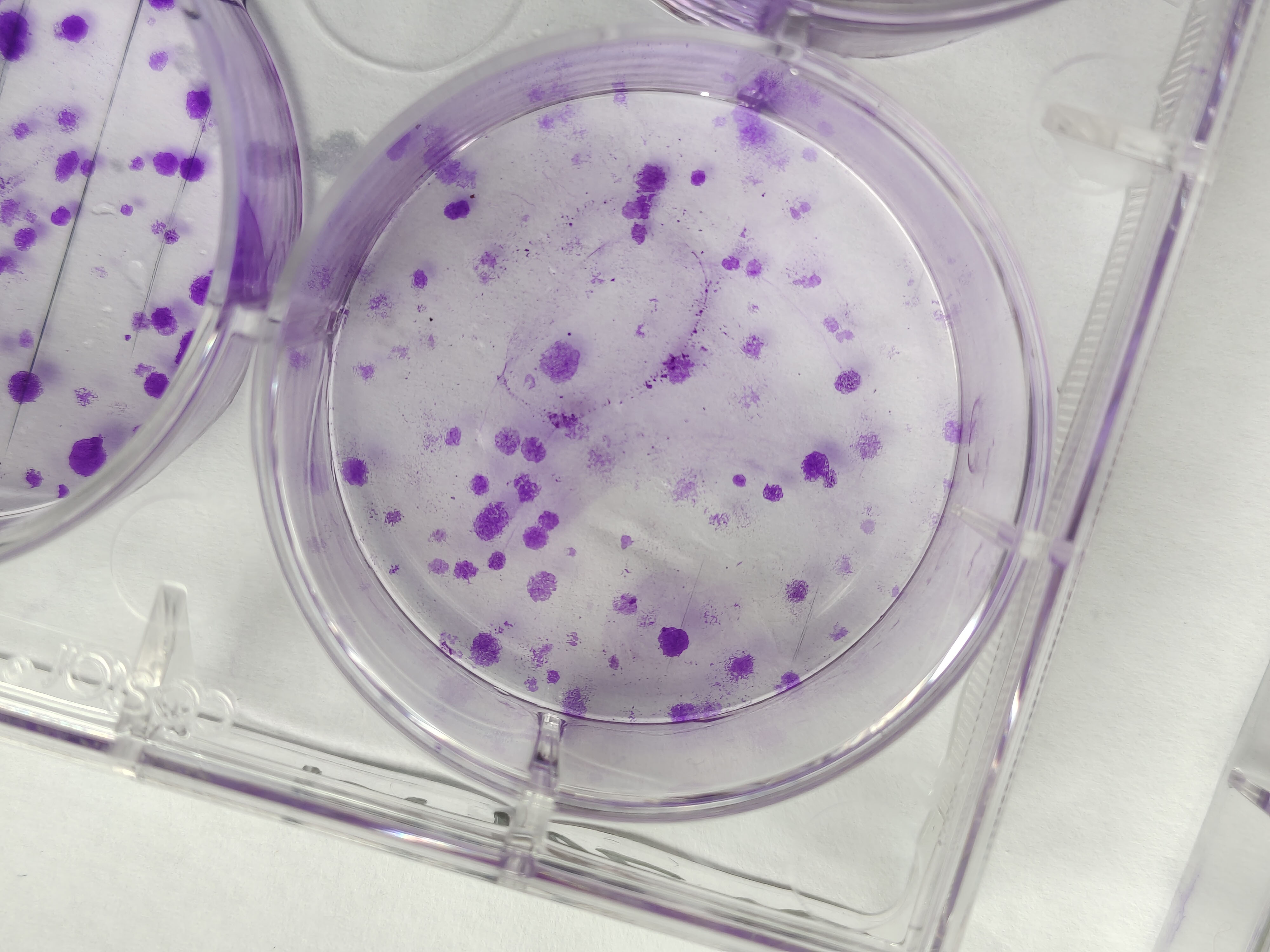

Supplement: Data S1 [file peerj-10-13871-s008.zip › source data/cell assay/Colony formation assay/H1299/H1299 NC/1299 bz nc3 6.26.jpg]

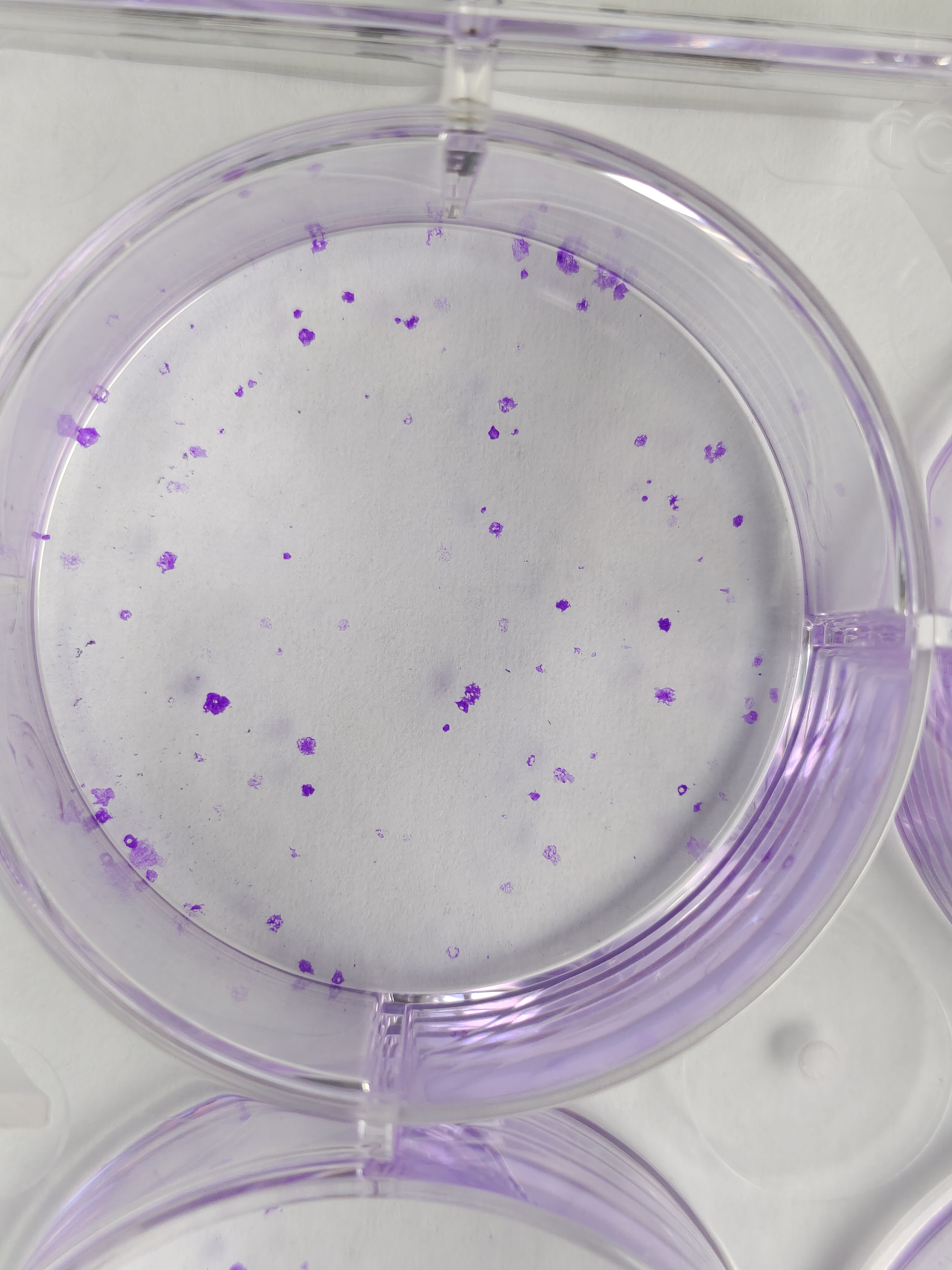

Supplement: Data S1 [file peerj-10-13871-s008.zip › source data/cell assay/Colony formation assay/HCC827/HCC827 BZRAP1-AS1 OE/1.jpg]

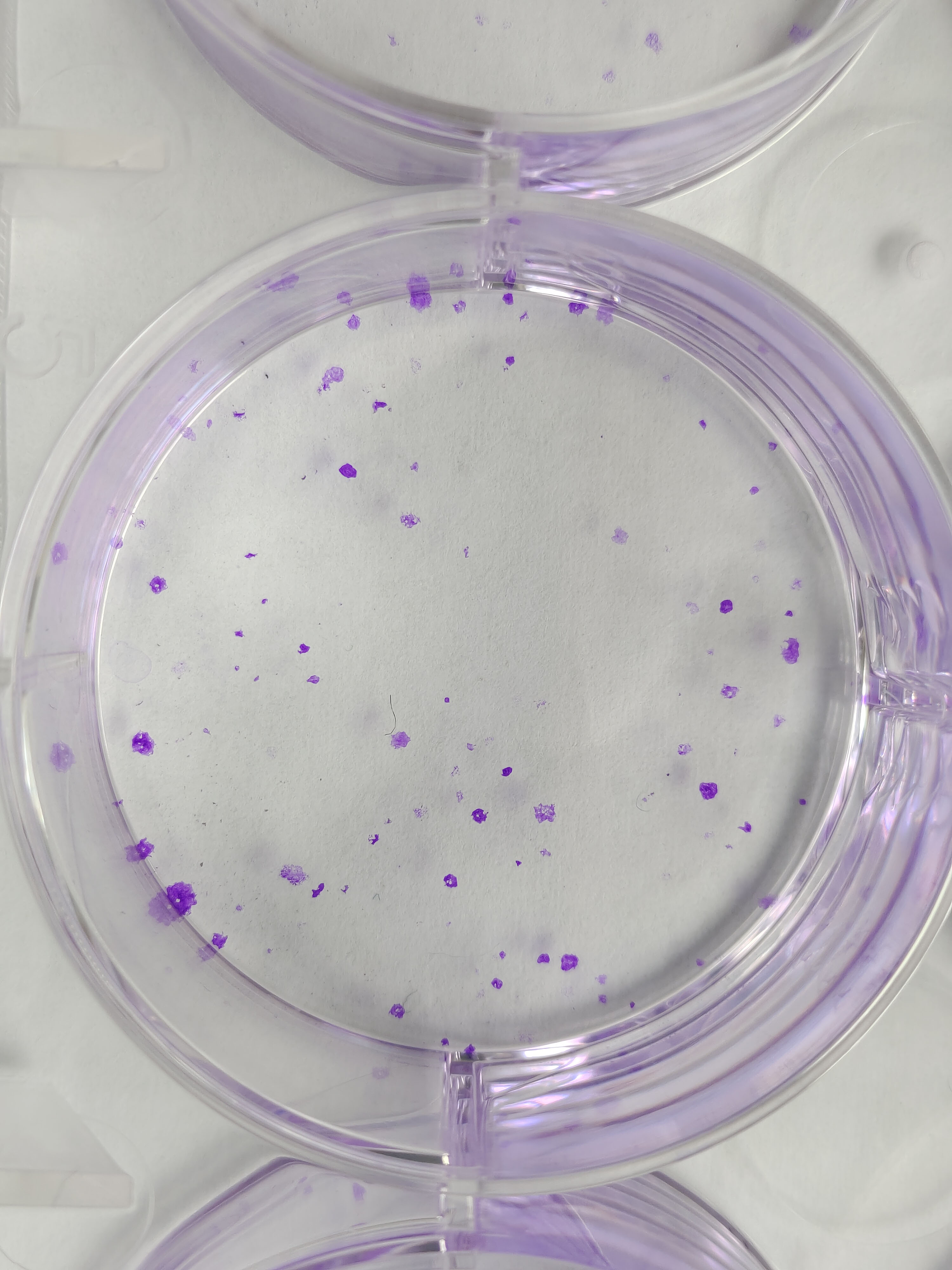

Supplement: Data S1 [file peerj-10-13871-s008.zip › source data/cell assay/Colony formation assay/HCC827/HCC827 BZRAP1-AS1 OE/2.jpg]

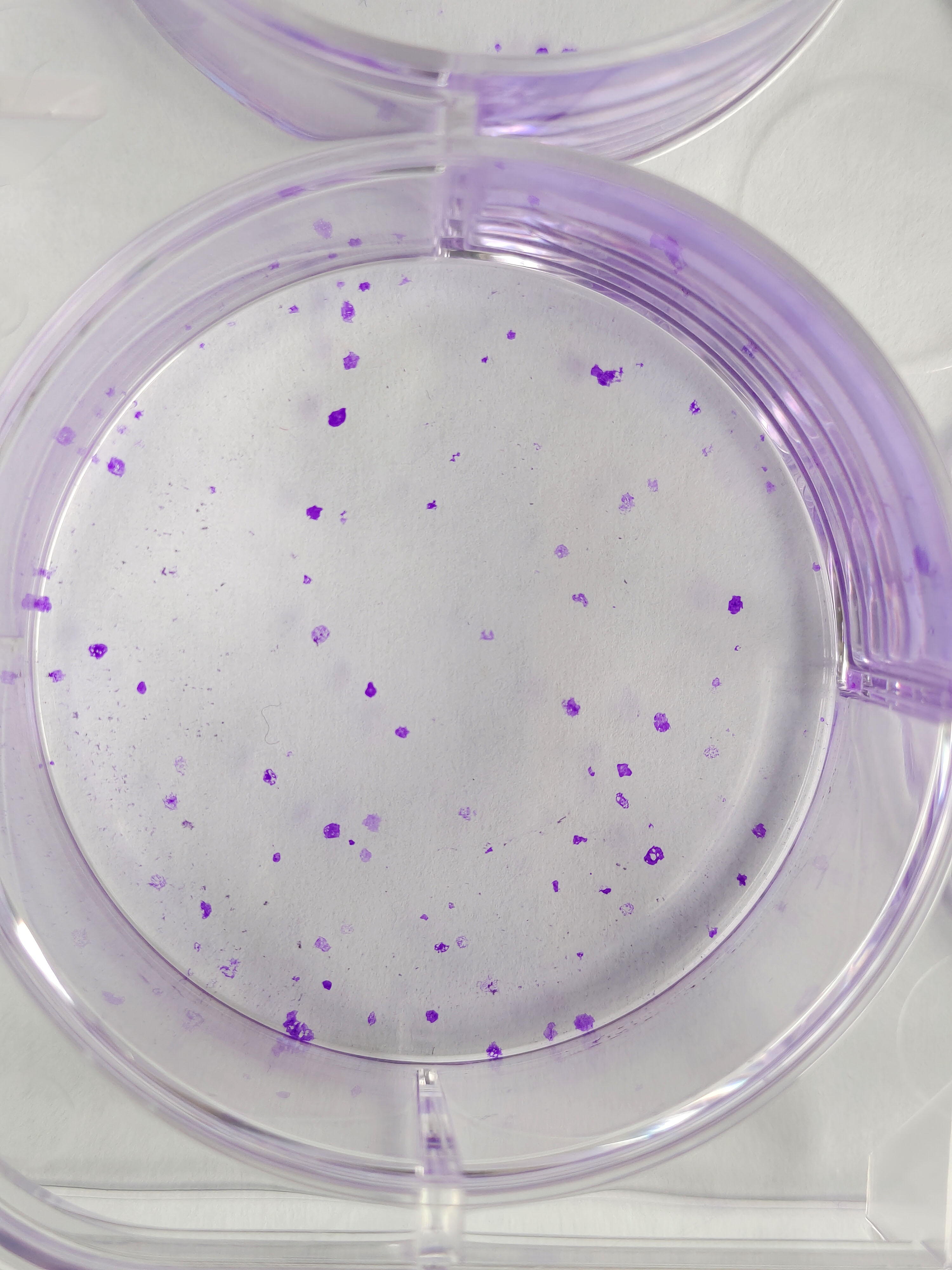

Supplement: Data S1 [file peerj-10-13871-s008.zip › source data/cell assay/Colony formation assay/HCC827/HCC827 BZRAP1-AS1 OE/3.jpg]

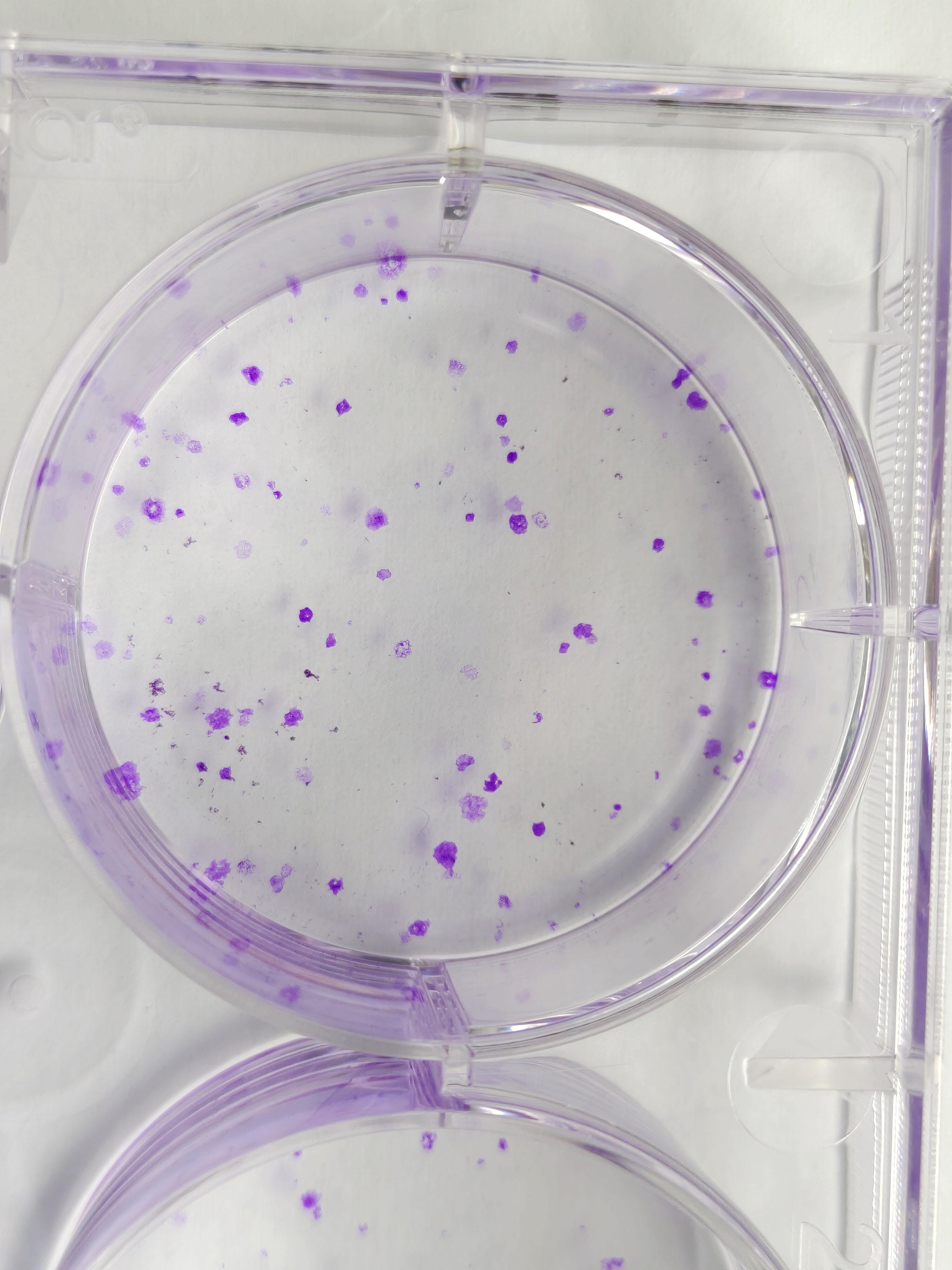

Supplement: Data S1 [file peerj-10-13871-s008.zip › source data/cell assay/Colony formation assay/HCC827/HCC827 NC/NC1.jpg]

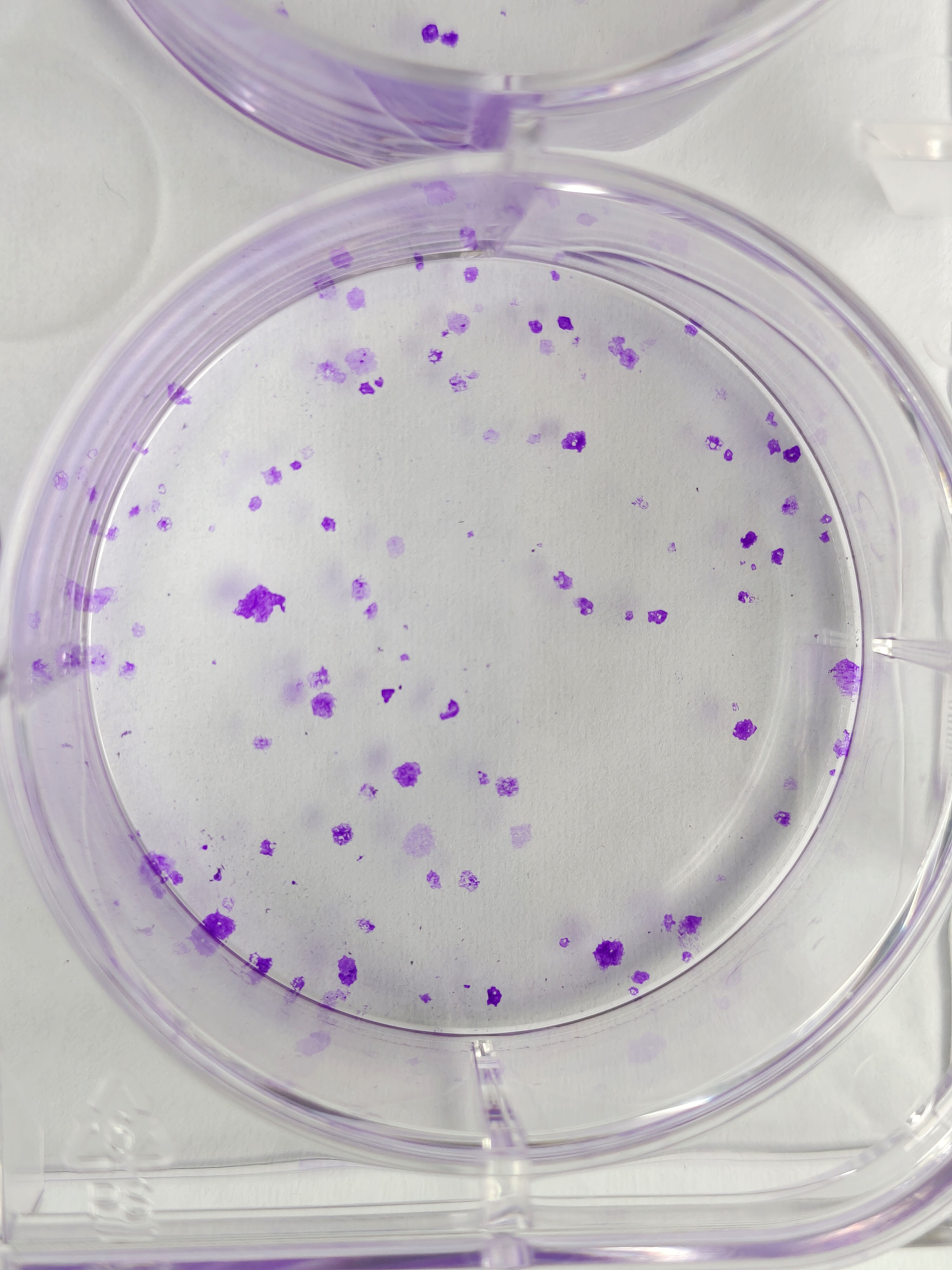

Supplement: Data S1 [file peerj-10-13871-s008.zip › source data/cell assay/Colony formation assay/HCC827/HCC827 NC/NC2.jpg]

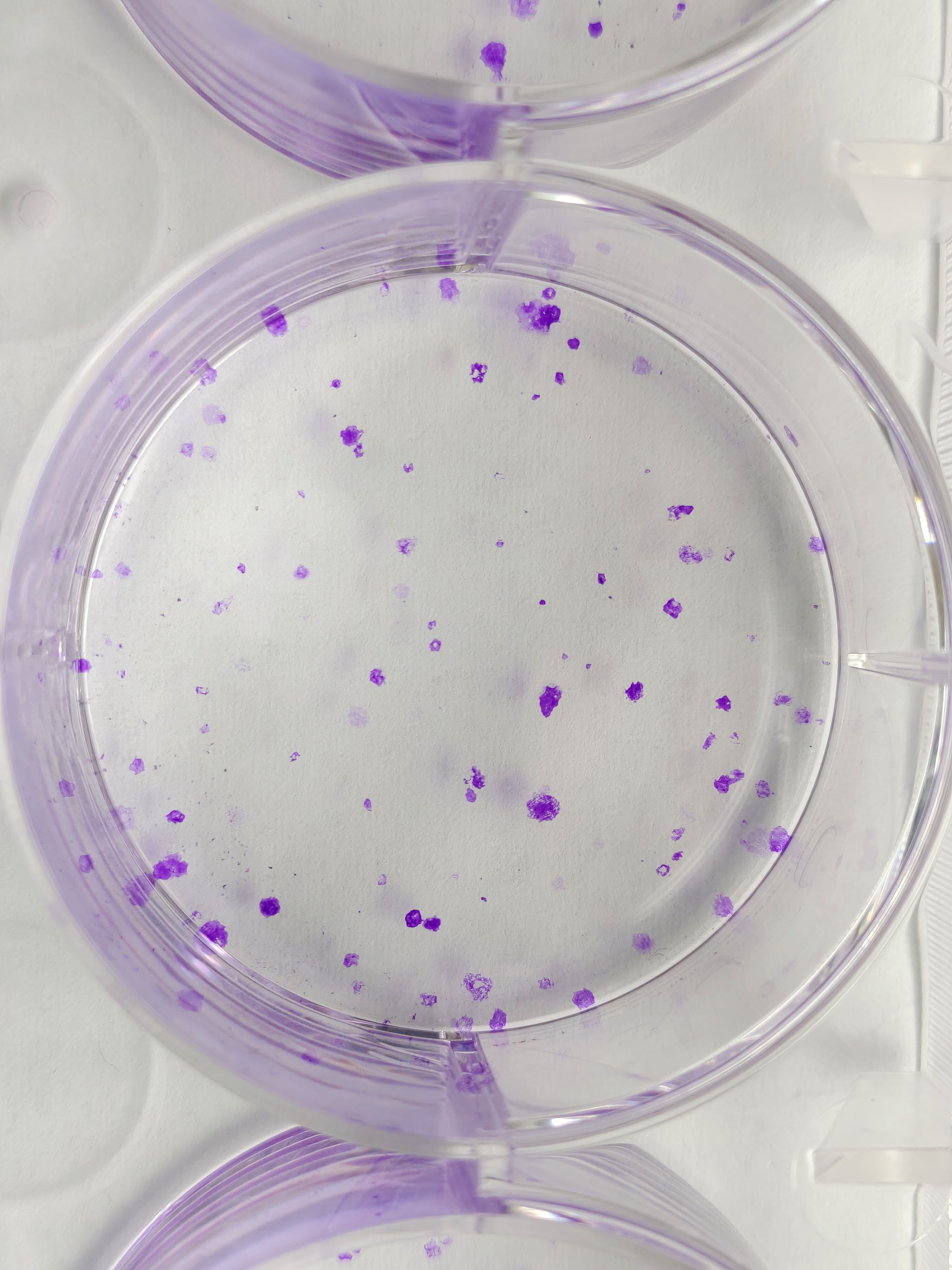

Supplement: Data S1 [file peerj-10-13871-s008.zip › source data/cell assay/Colony formation assay/HCC827/HCC827 NC/NC3.jpg]

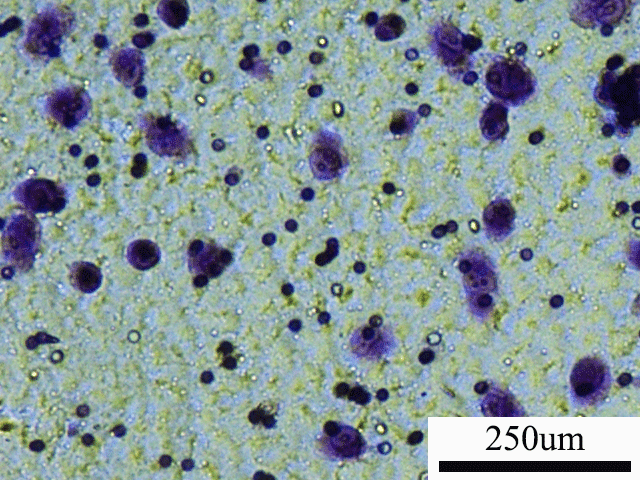

Supplement: Data S1 [file peerj-10-13871-s008.zip › source data/cell assay/transwell assay/H1299/H1299 BZRAP1-AS1 OE/4K-New-project_1299bz-oe10_ch001_01.gif]

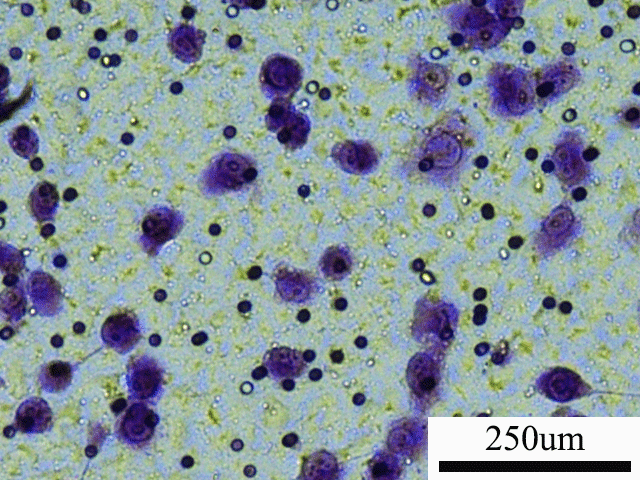

Supplement: Data S1 [file peerj-10-13871-s008.zip › source data/cell assay/transwell assay/H1299/H1299 BZRAP1-AS1 OE/4K-New-project_1299bz-oe10_ch001_02.gif]

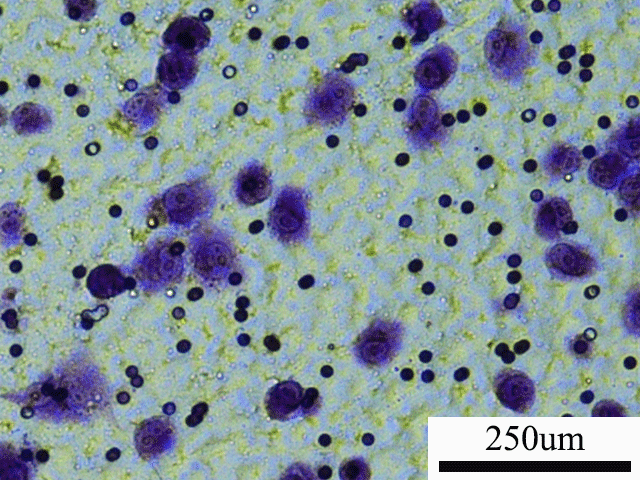

Supplement: Data S1 [file peerj-10-13871-s008.zip › source data/cell assay/transwell assay/H1299/H1299 BZRAP1-AS1 OE/4K-New-project_1299bz-oe10_ch001_03.gif]

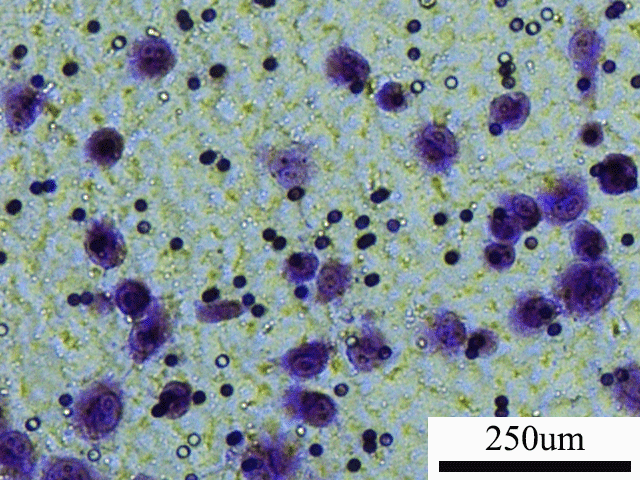

Supplement: Data S1 [file peerj-10-13871-s008.zip › source data/cell assay/transwell assay/H1299/H1299 BZRAP1-AS1 OE/4K-New-project_1299bz-oe10_ch001_04.gif]

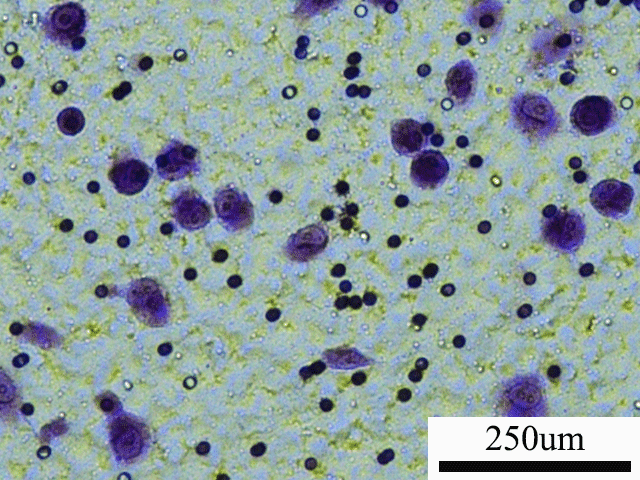

Supplement: Data S1 [file peerj-10-13871-s008.zip › source data/cell assay/transwell assay/H1299/H1299 BZRAP1-AS1 OE/4K-New-project_1299bz-oe10_ch001_05.gif]

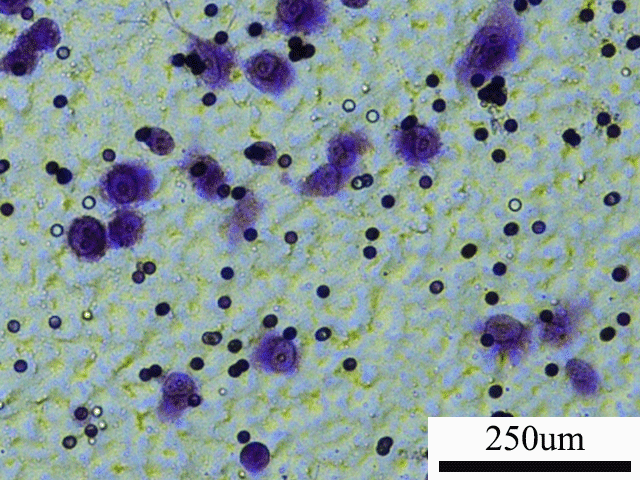

Supplement: Data S1 [file peerj-10-13871-s008.zip › source data/cell assay/transwell assay/H1299/H1299 BZRAP1-AS1 OE/4K-New-project_1299bz-oe10_ch001_06.gif]

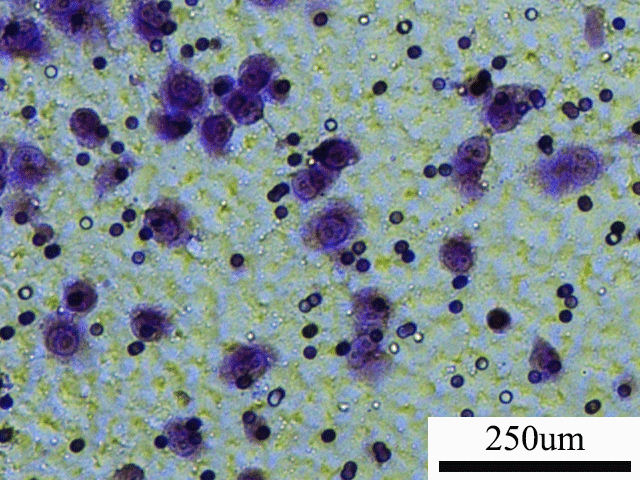

Supplement: Data S1 [file peerj-10-13871-s008.zip › source data/cell assay/transwell assay/H1299/H1299 BZRAP1-AS1 OE/4K-New-project_1299bz-oe10_ch001_07.gif]

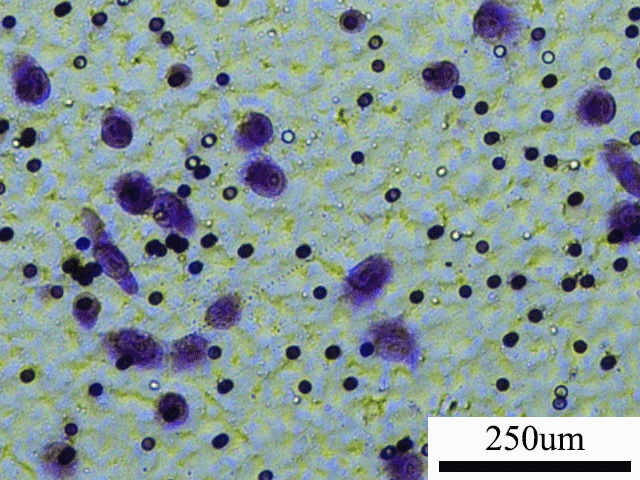

Supplement: Data S1 [file peerj-10-13871-s008.zip › source data/cell assay/transwell assay/H1299/H1299 BZRAP1-AS1 OE/4K-New-project_1299bz-oe10_ch001_08.gif]

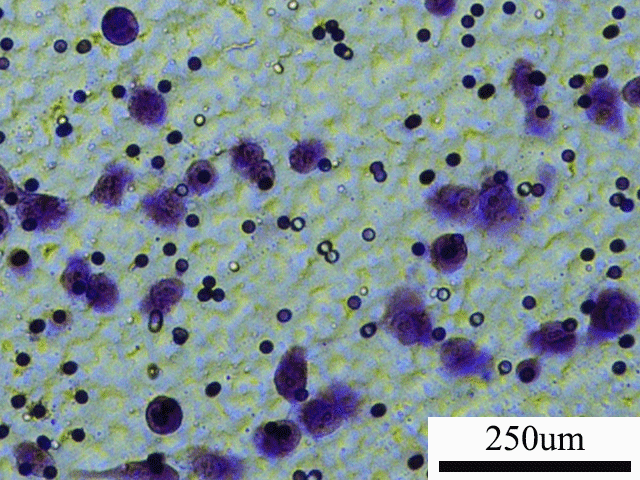

Supplement: Data S1 [file peerj-10-13871-s008.zip › source data/cell assay/transwell assay/H1299/H1299 BZRAP1-AS1 OE/4K-New-project_1299bz-oe10_ch001_09.gif]

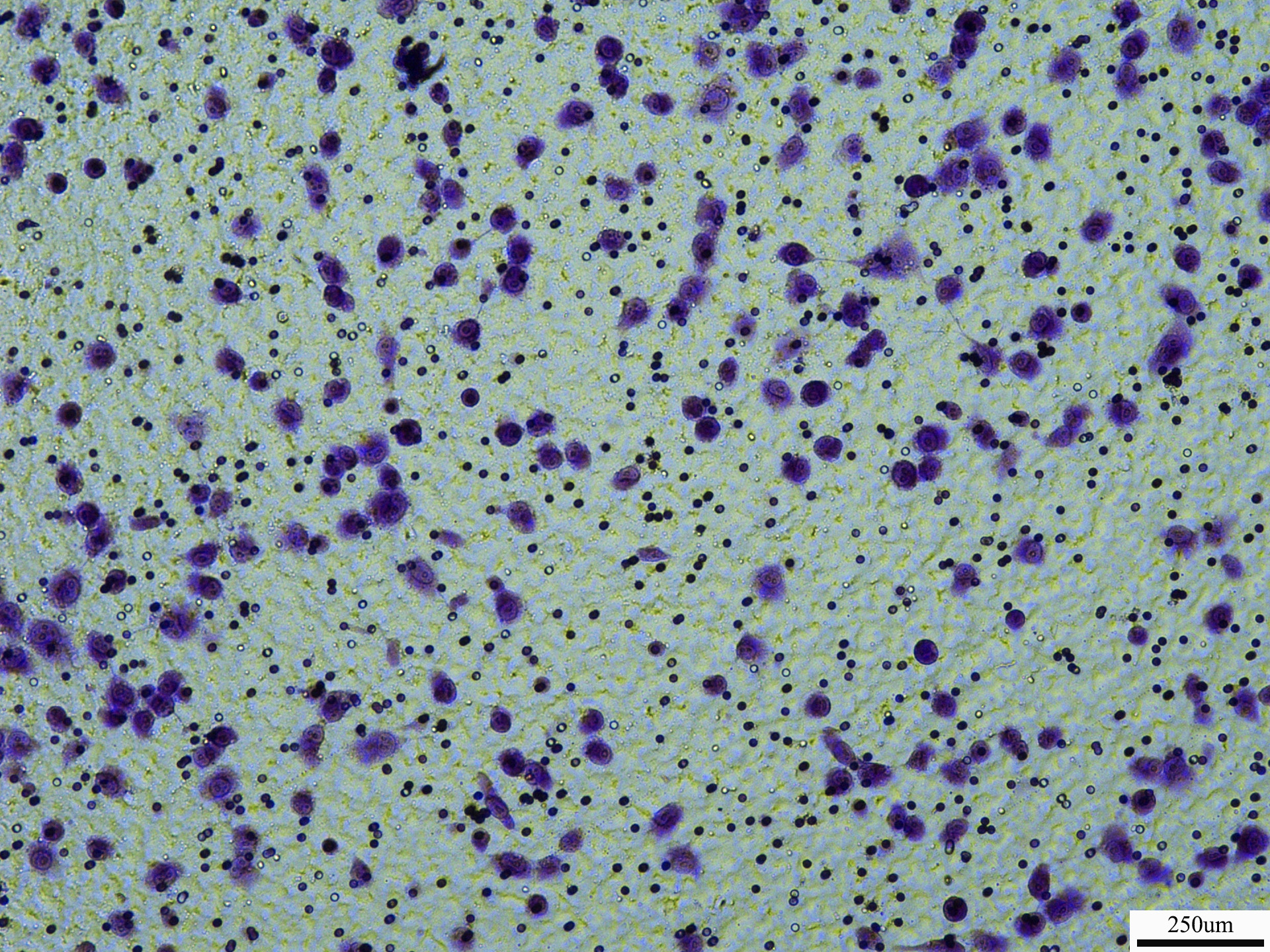

Supplement: Data S1 [file peerj-10-13871-s008.zip › source data/cell assay/transwell assay/H1299/H1299 BZRAP1-AS1 OE/H1299 BZRAP1-AS1 OE(X10).jpg]

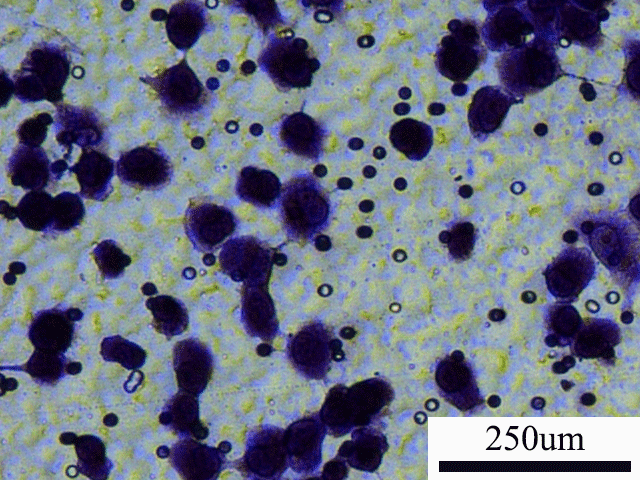

Supplement: Data S1 [file peerj-10-13871-s008.zip › source data/cell assay/transwell assay/H1299/H1299-NC/4J-New-project_1299-bz10-nc_ch001_01.gif]

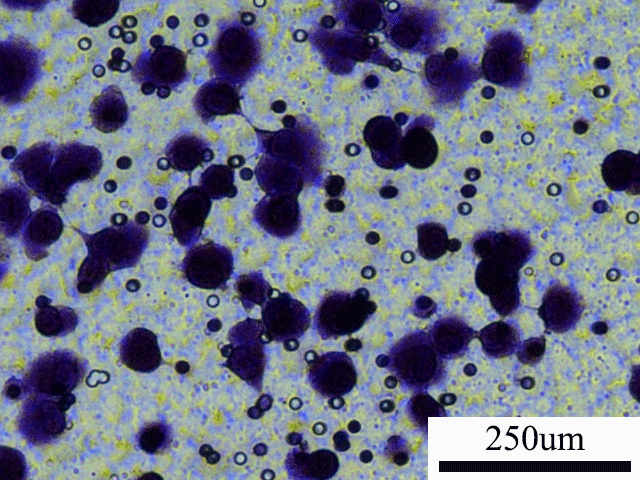

Supplement: Data S1 [file peerj-10-13871-s008.zip › source data/cell assay/transwell assay/H1299/H1299-NC/4J-New-project_1299-bz10-nc_ch001_02.gif]

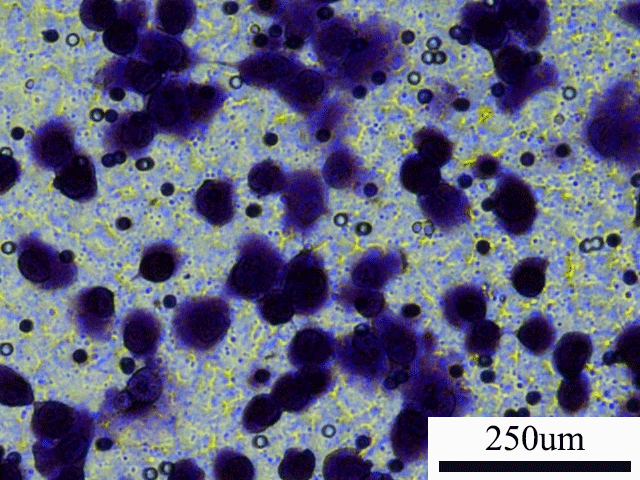

Supplement: Data S1 [file peerj-10-13871-s008.zip › source data/cell assay/transwell assay/H1299/H1299-NC/4J-New-project_1299-bz10-nc_ch001_03.gif]

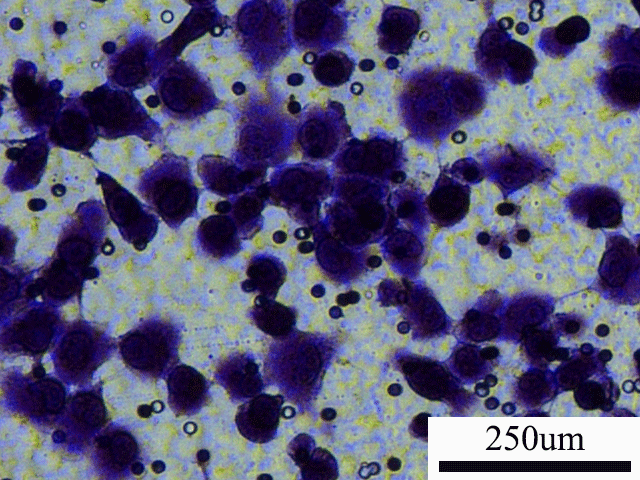

Supplement: Data S1 [file peerj-10-13871-s008.zip › source data/cell assay/transwell assay/H1299/H1299-NC/4J-New-project_1299-bz10-nc_ch001_04.gif]

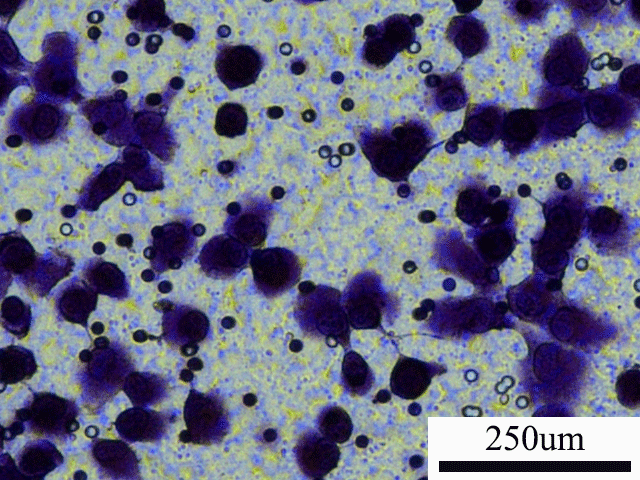

Supplement: Data S1 [file peerj-10-13871-s008.zip › source data/cell assay/transwell assay/H1299/H1299-NC/4J-New-project_1299-bz10-nc_ch001_05.gif]

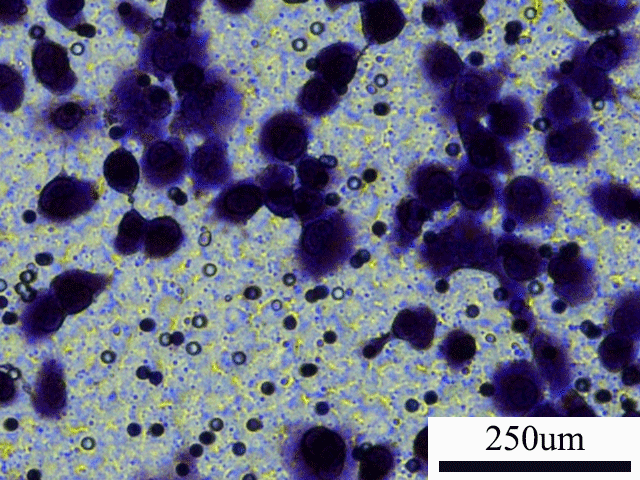

Supplement: Data S1 [file peerj-10-13871-s008.zip › source data/cell assay/transwell assay/H1299/H1299-NC/4J-New-project_1299-bz10-nc_ch001_06.gif]

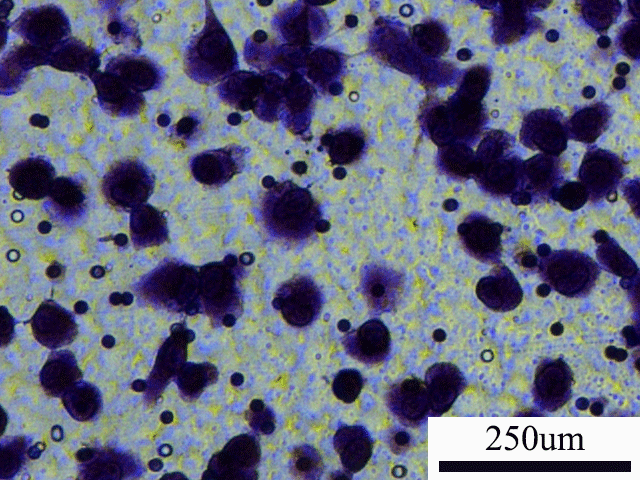

Supplement: Data S1 [file peerj-10-13871-s008.zip › source data/cell assay/transwell assay/H1299/H1299-NC/4J-New-project_1299-bz10-nc_ch001_07.gif]

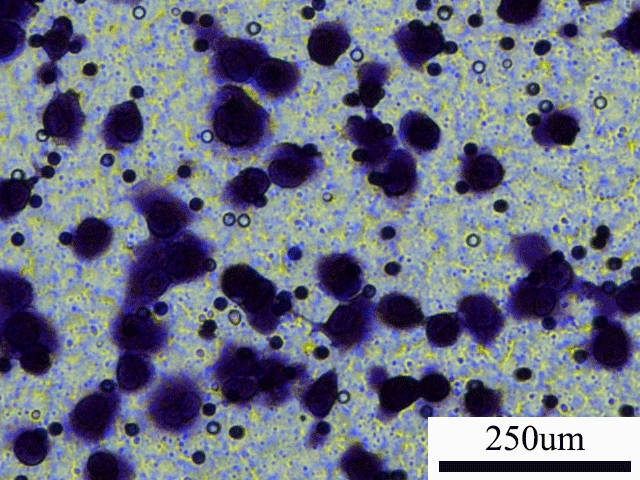

Supplement: Data S1 [file peerj-10-13871-s008.zip › source data/cell assay/transwell assay/H1299/H1299-NC/4J-New-project_1299-bz10-nc_ch001_08.gif]

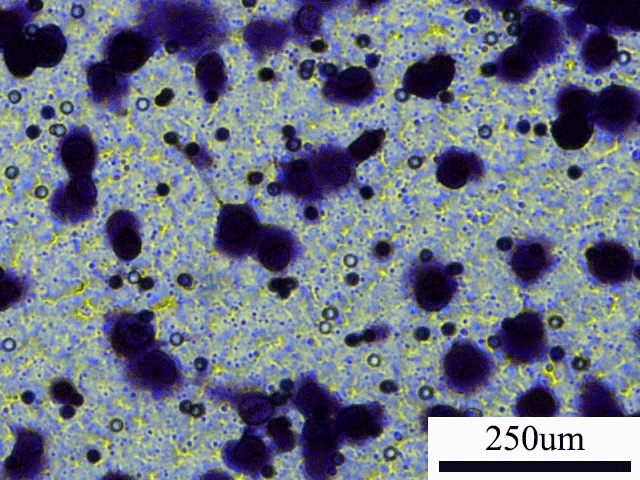

Supplement: Data S1 [file peerj-10-13871-s008.zip › source data/cell assay/transwell assay/H1299/H1299-NC/4J-New-project_1299-bz10-nc_ch001_09.gif]

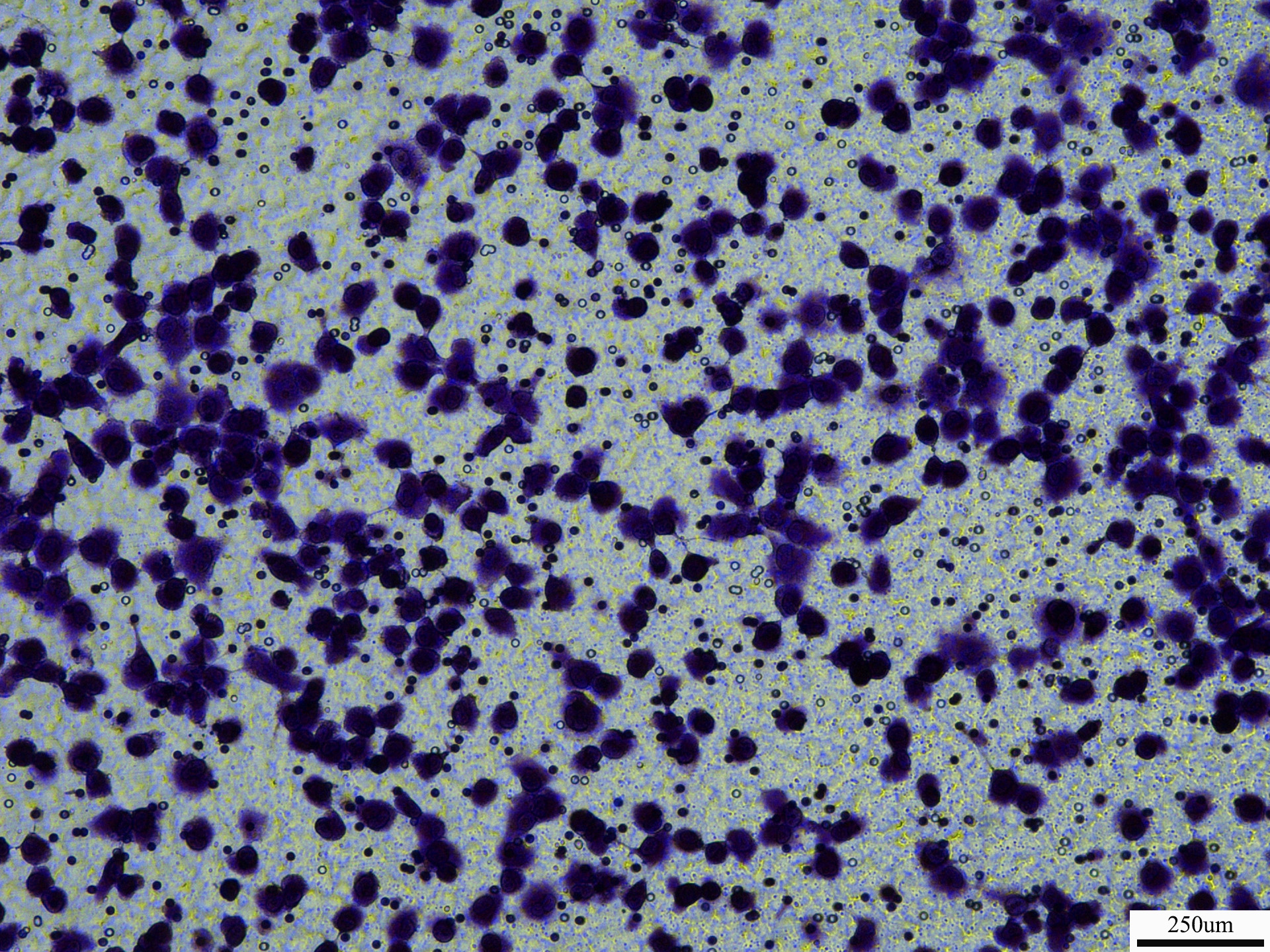

Supplement: Data S1 [file peerj-10-13871-s008.zip › source data/cell assay/transwell assay/H1299/H1299-NC/H1299 NC(X10).jpg]

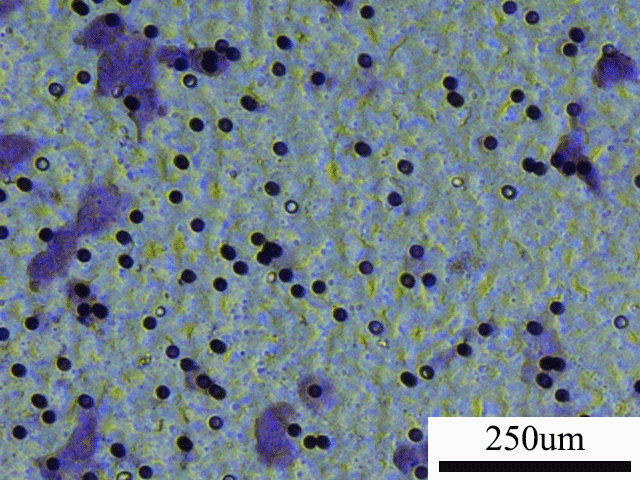

Supplement: Data S1 [file peerj-10-13871-s008.zip › source data/cell assay/transwell assay/HCC827/HCC827 BZRAP1-AS1 OE/4M-New-project_hcc827-bz-oe10_ch001_01.gif]

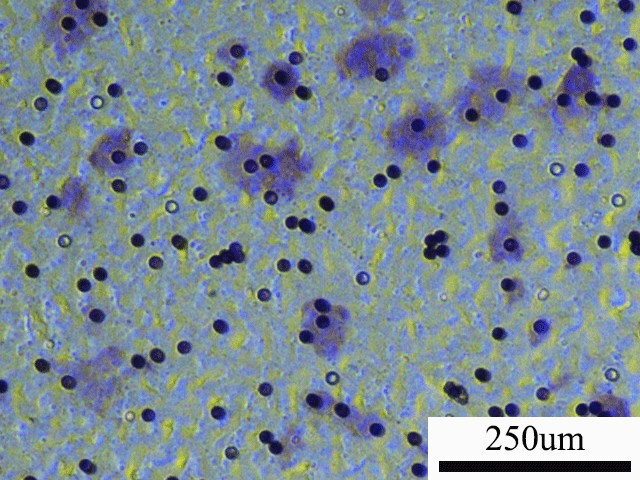

Supplement: Data S1 [file peerj-10-13871-s008.zip › source data/cell assay/transwell assay/HCC827/HCC827 BZRAP1-AS1 OE/4M-New-project_hcc827-bz-oe10_ch001_02.gif]

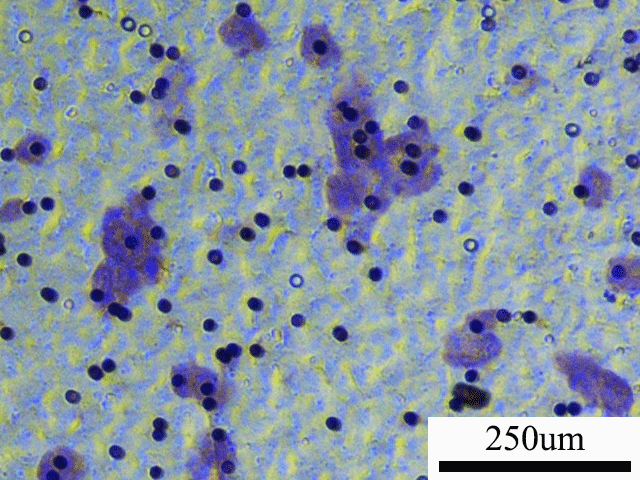

Supplement: Data S1 [file peerj-10-13871-s008.zip › source data/cell assay/transwell assay/HCC827/HCC827 BZRAP1-AS1 OE/4M-New-project_hcc827-bz-oe10_ch001_03.gif]

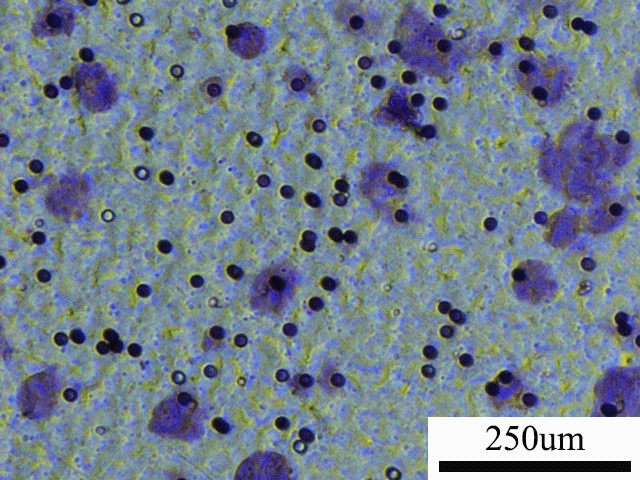

Supplement: Data S1 [file peerj-10-13871-s008.zip › source data/cell assay/transwell assay/HCC827/HCC827 BZRAP1-AS1 OE/4M-New-project_hcc827-bz-oe10_ch001_04.gif]

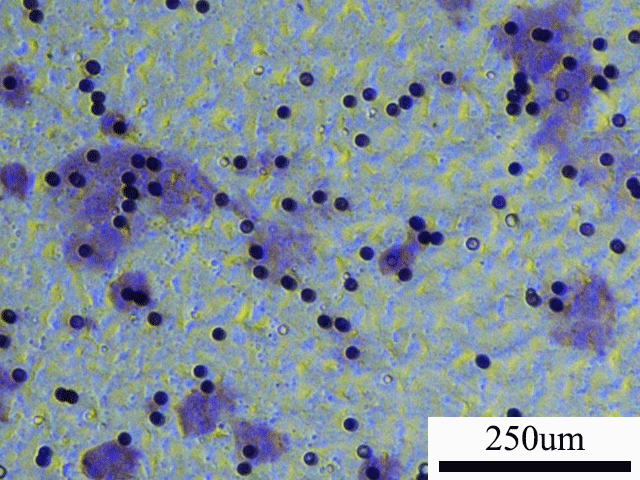

Supplement: Data S1 [file peerj-10-13871-s008.zip › source data/cell assay/transwell assay/HCC827/HCC827 BZRAP1-AS1 OE/4M-New-project_hcc827-bz-oe10_ch001_05.gif]

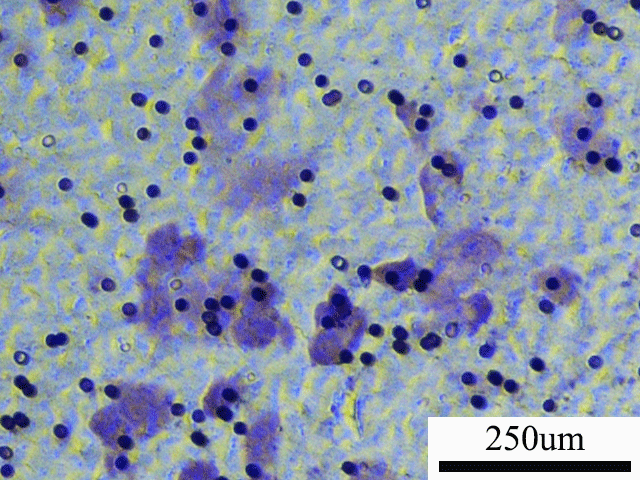

Supplement: Data S1 [file peerj-10-13871-s008.zip › source data/cell assay/transwell assay/HCC827/HCC827 BZRAP1-AS1 OE/4M-New-project_hcc827-bz-oe10_ch001_06.gif]

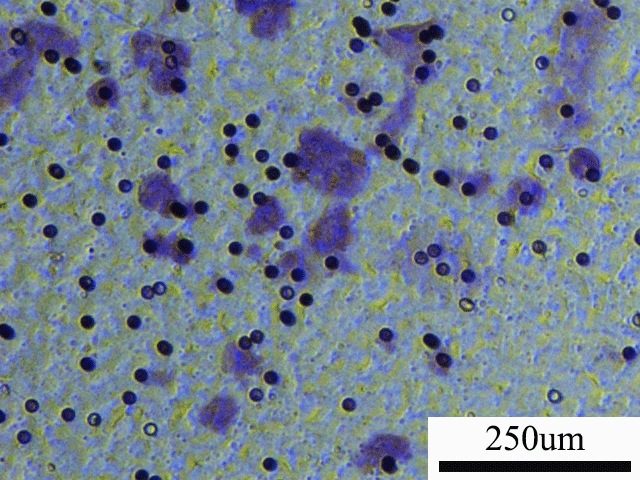

Supplement: Data S1 [file peerj-10-13871-s008.zip › source data/cell assay/transwell assay/HCC827/HCC827 BZRAP1-AS1 OE/4M-New-project_hcc827-bz-oe10_ch001_07.gif]

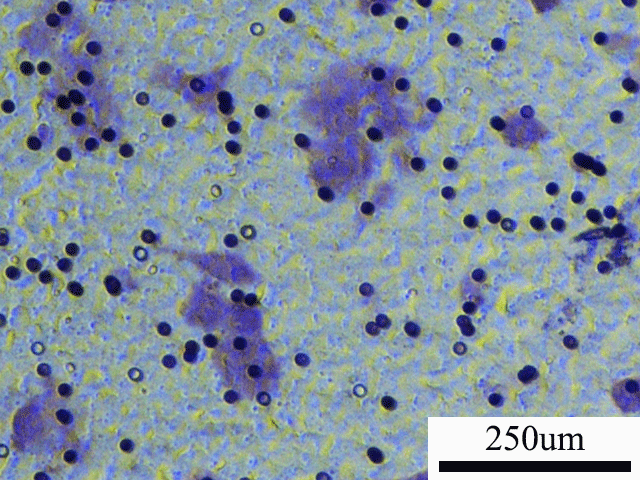

Supplement: Data S1 [file peerj-10-13871-s008.zip › source data/cell assay/transwell assay/HCC827/HCC827 BZRAP1-AS1 OE/4M-New-project_hcc827-bz-oe10_ch001_08.gif]

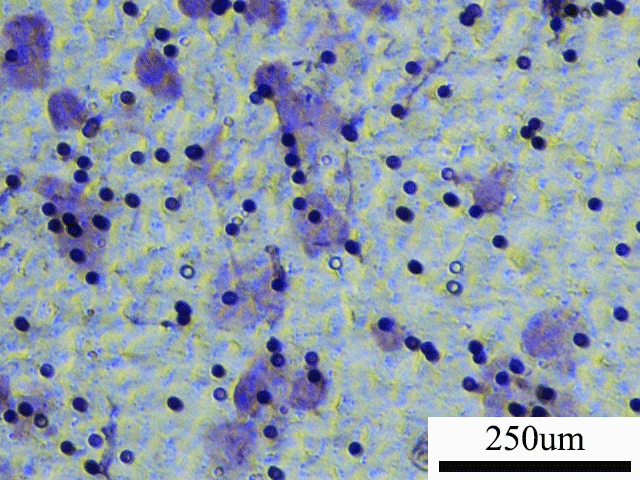

Supplement: Data S1 [file peerj-10-13871-s008.zip › source data/cell assay/transwell assay/HCC827/HCC827 BZRAP1-AS1 OE/4M-New-project_hcc827-bz-oe10_ch001_09.gif]

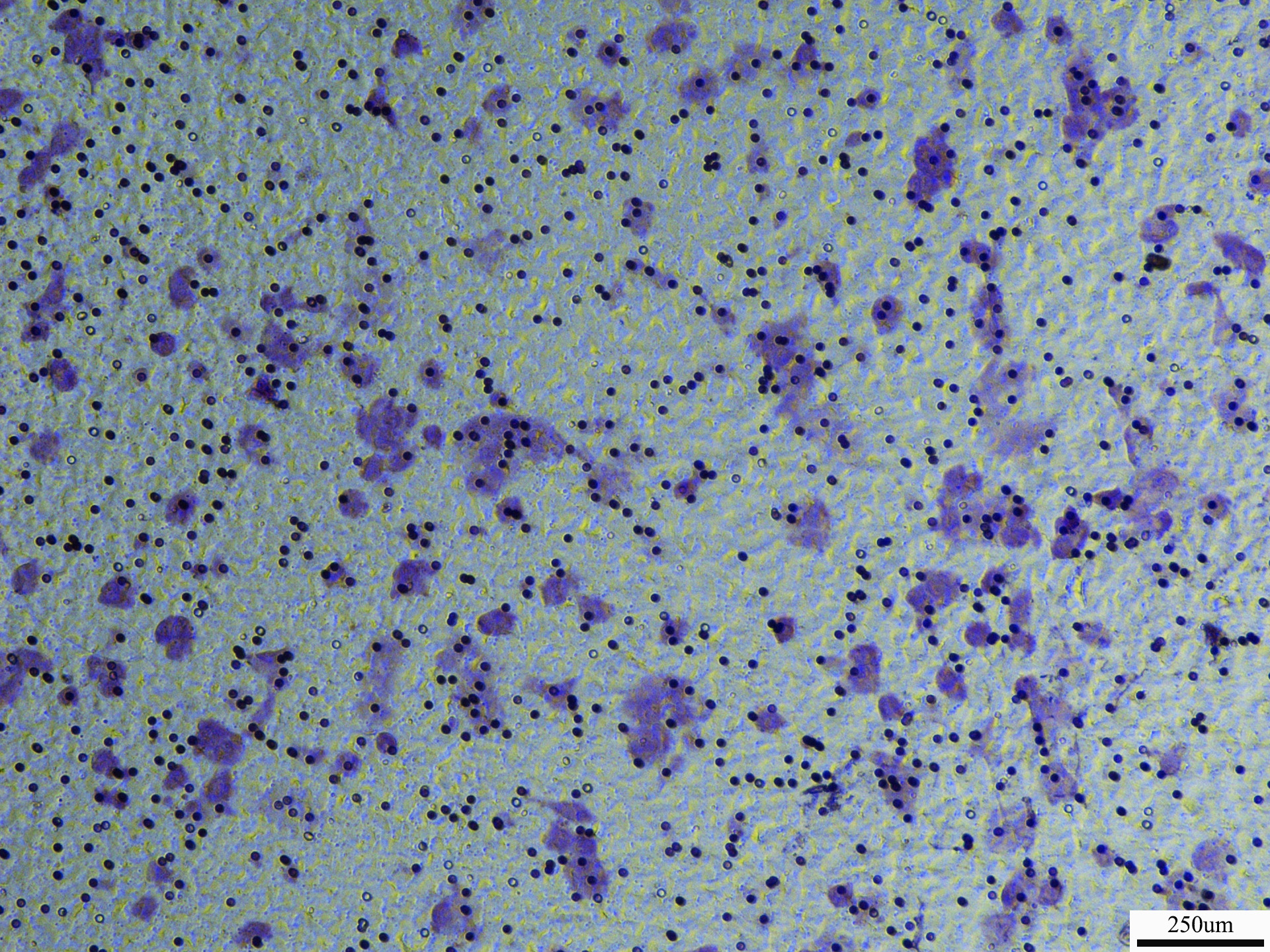

Supplement: Data S1 [file peerj-10-13871-s008.zip › source data/cell assay/transwell assay/HCC827/HCC827 BZRAP1-AS1 OE/HCC827 BZRAP1-AS1 OE.jpg]

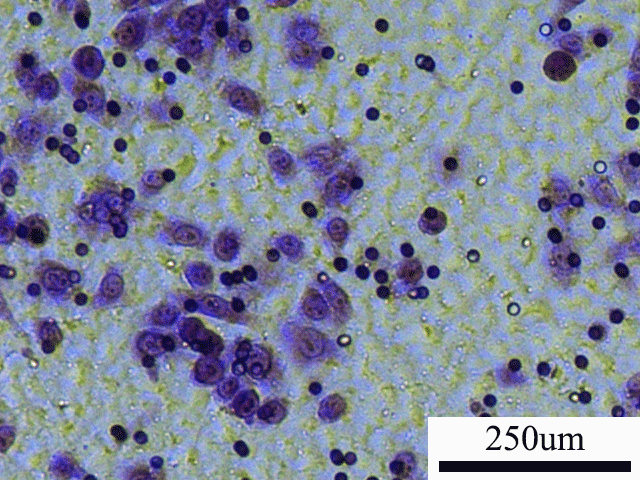

Supplement: Data S1 [file peerj-10-13871-s008.zip › source data/cell assay/transwell assay/HCC827/HCC827 NC/4L-New-project_hcc827-bz-nc10_ch001_01.gif]

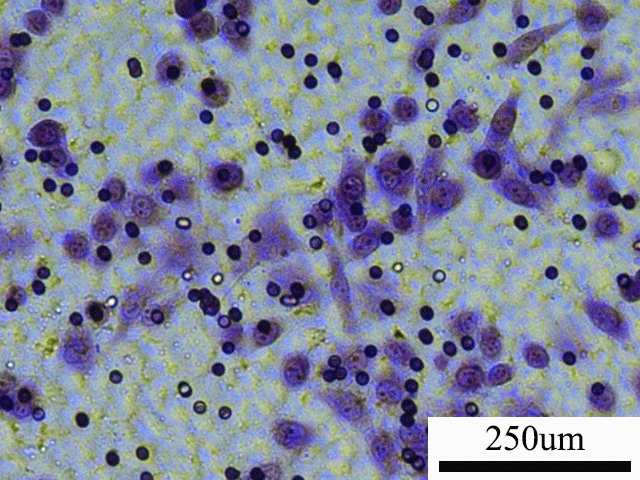

Supplement: Data S1 [file peerj-10-13871-s008.zip › source data/cell assay/transwell assay/HCC827/HCC827 NC/4L-New-project_hcc827-bz-nc10_ch001_02.gif]

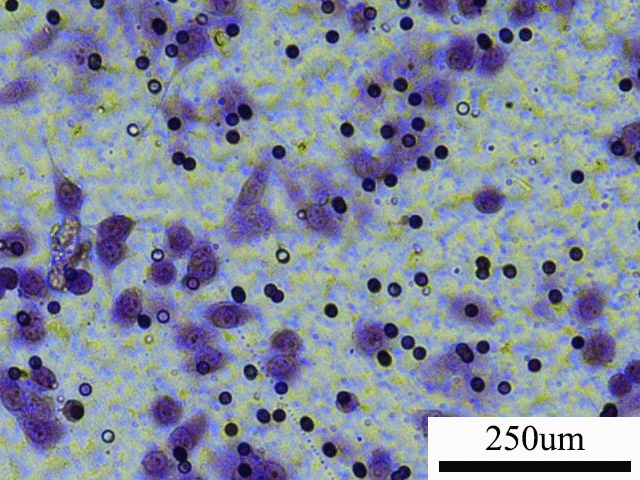

Supplement: Data S1 [file peerj-10-13871-s008.zip › source data/cell assay/transwell assay/HCC827/HCC827 NC/4L-New-project_hcc827-bz-nc10_ch001_03.gif]

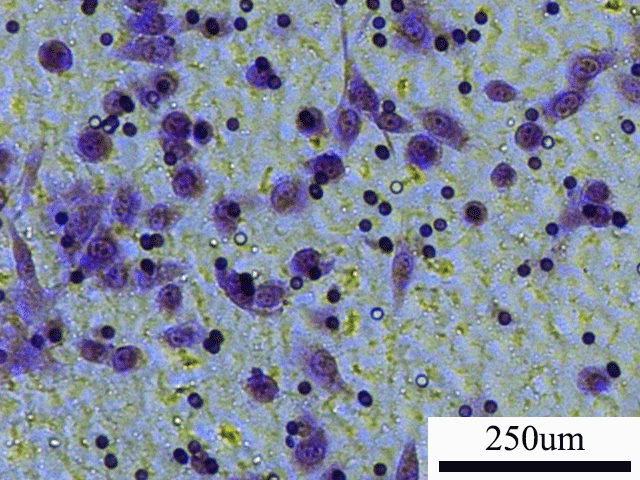

Supplement: Data S1 [file peerj-10-13871-s008.zip › source data/cell assay/transwell assay/HCC827/HCC827 NC/4L-New-project_hcc827-bz-nc10_ch001_04.gif]

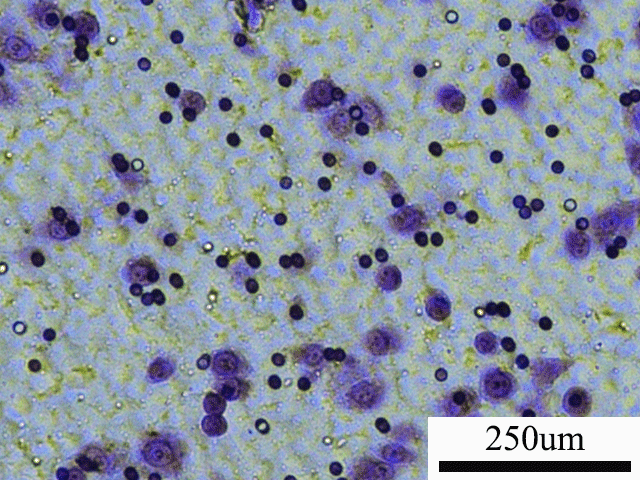

Supplement: Data S1 [file peerj-10-13871-s008.zip › source data/cell assay/transwell assay/HCC827/HCC827 NC/4L-New-project_hcc827-bz-nc10_ch001_05.gif]

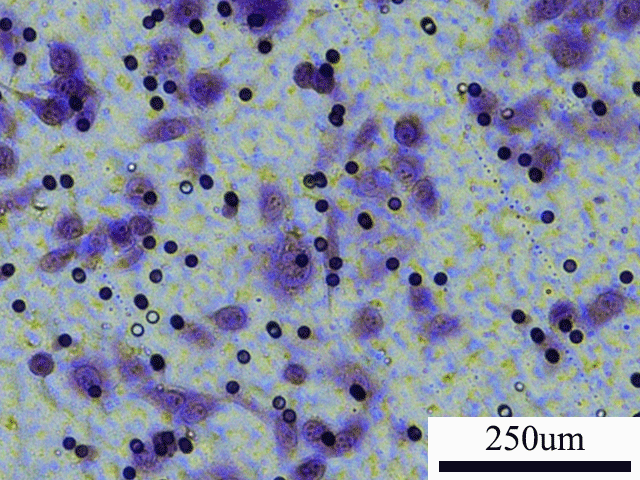

Supplement: Data S1 [file peerj-10-13871-s008.zip › source data/cell assay/transwell assay/HCC827/HCC827 NC/4L-New-project_hcc827-bz-nc10_ch001_06.gif]

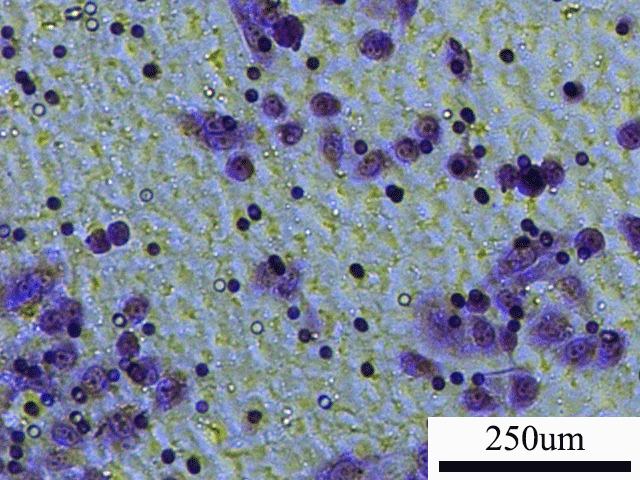

Supplement: Data S1 [file peerj-10-13871-s008.zip › source data/cell assay/transwell assay/HCC827/HCC827 NC/4L-New-project_hcc827-bz-nc10_ch001_07.gif]

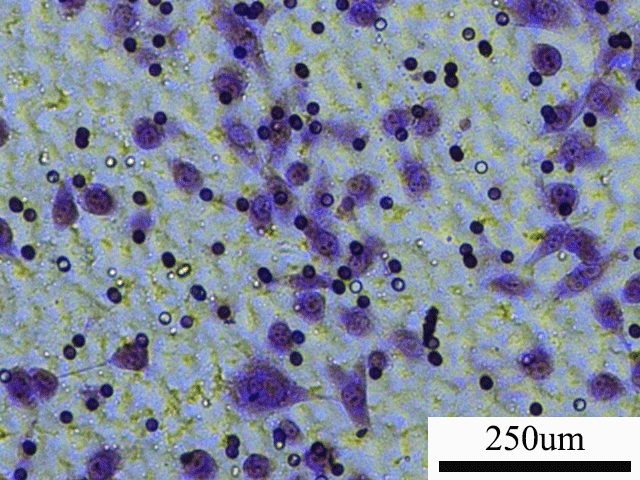

Supplement: Data S1 [file peerj-10-13871-s008.zip › source data/cell assay/transwell assay/HCC827/HCC827 NC/4L-New-project_hcc827-bz-nc10_ch001_08.gif]

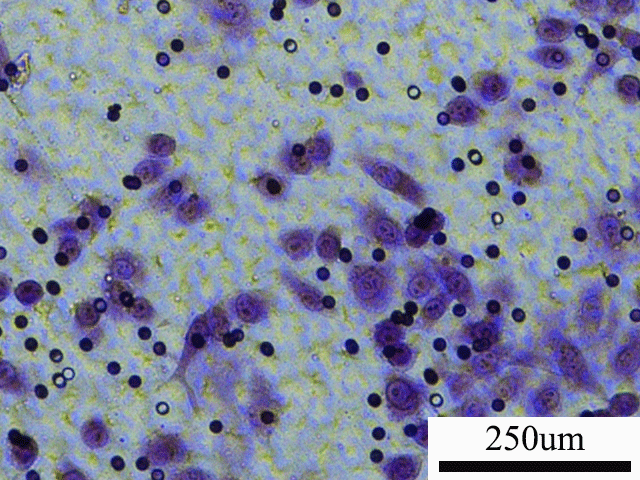

Supplement: Data S1 [file peerj-10-13871-s008.zip › source data/cell assay/transwell assay/HCC827/HCC827 NC/4L-New-project_hcc827-bz-nc10_ch001_09.gif]

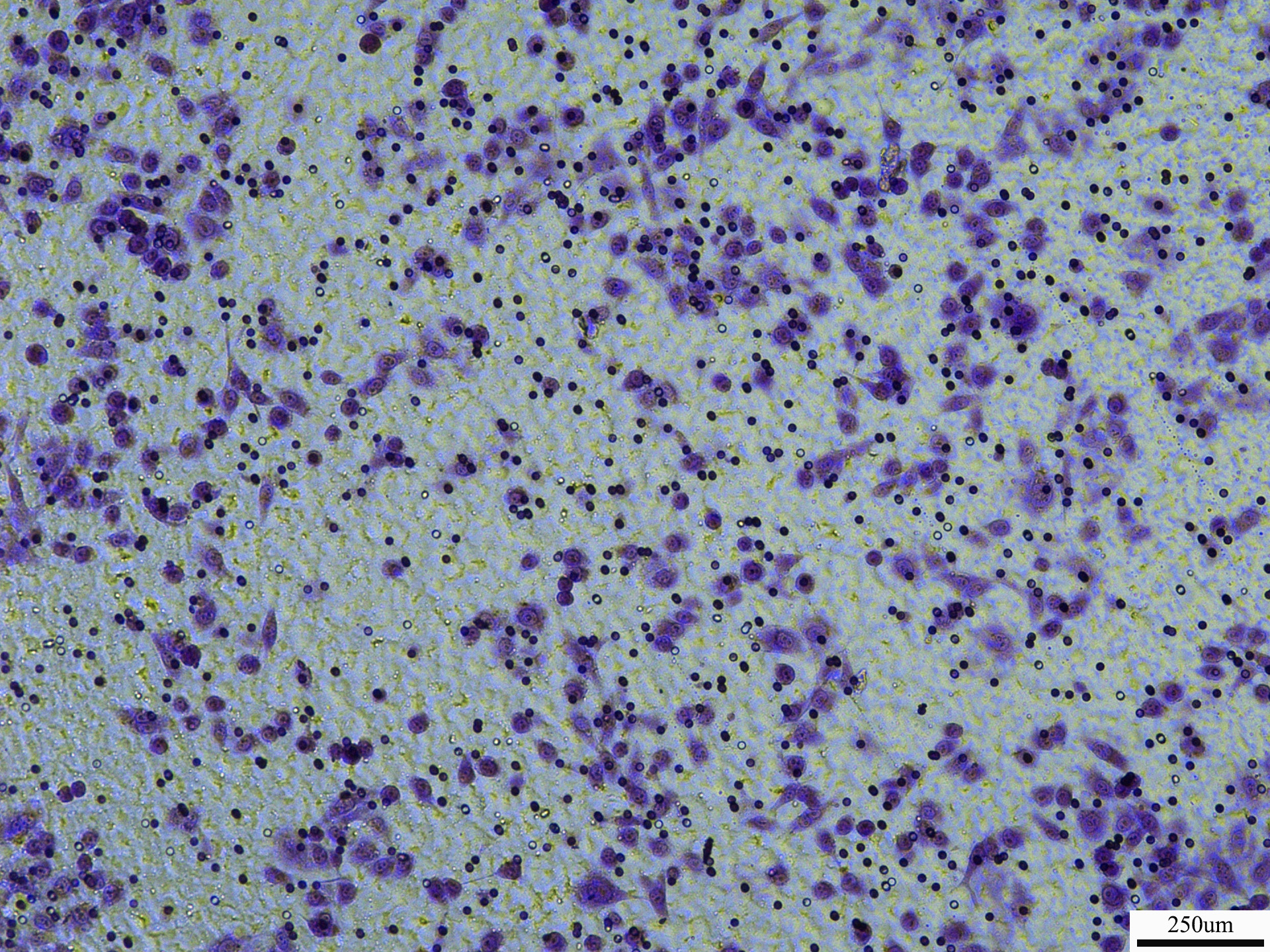

Supplement: Data S1 [file peerj-10-13871-s008.zip › source data/cell assay/transwell assay/HCC827/HCC827 NC/HCC827 NC (X10).jpg]
