# Supplementary material for: Long non-coding RNA BZRAP1-AS1 functions in malignancy and prognosis for non-small-cell lung cancer
Source: PeerJ. 2022 Aug 23;10:e13871. doi: 10.7717/peerj.13871 (PMC9415519; doi:10.7717/peerj.13871)
Supplement: Table S1 [file peerj-10-13871-s004.docx]

Supplementary Tables1 The proportion of smoking patients in sex and histologic type

|  | **Smokers** | **Nonsmokers** |
| --- | --- | --- |
| **Sex** |  |  |
| Male | 40(87.0%) | 6(13.0) |
| Female | 1(5.9%) | 16(94.1) |
| **Histologic type** |  |  |
| LUAD | 19(54.3%) | 16(45.7%) |
| LUSC | 22(78.6%) | 6(21.4%) |
