# Supplementary material for: Long non-coding RNA BZRAP1-AS1 functions in malignancy and prognosis for non-small-cell lung cancer
Source: PeerJ. 2022 Aug 23;10:e13871. doi: 10.7717/peerj.13871 (PMC9415519; doi:10.7717/peerj.13871)
Supplement: Table S2 [file peerj-10-13871-s005.docx]

Supplementary Tables 2 Annotation information and expression changes of predicted target genes with the overexpression of *BZRAP1-AS1* (Mouse tumor tissue sequencing data).

| Gene | KEGG Pathway/GO annotation | adj.*P.*Val | FC | Regulation |
| --- | --- | --- | --- | --- |
| RPS6KA6 | mTOR signaling pathway | 1.5579E-08 | -5.612 | down |
| LUM | Proteoglycans in cancer | 9.4147E-07 | -3.493 | down |
| TWIST1 | Proteoglycans in cancer | 1.9377E-06 | -5.455 | down |
| PRKACB | Wnt signaling pathway, Proteoglycans in cancer | 4.1234E-05 | -2.733 | down |
| TWIST2 | Proteoglycans in cancer | 1.8585E-04 | -3.256 | down |
| PPM1A | MAPK signaling pathway | 2.3350E-09 | 2.070 | up |
| FGF14 | MAPK signaling pathway | 1.4308E-05 | 2.747 | up |
| CNTN1 | CAMs | 1.0246E-04 | 3.691 | up |
| L1CAM | CAMs | 1.4256E-04 | 4.906 | up |
| VDR | negative regulation of cell proliferation | 1.4316E-04 | 2.383 | up |
| DPYSL3 | negative regulation of cell migration | 6.77E-03 | 2.072 | up |
| ARHGAP4 | negative regulation of cell migration | 1.57E-02 | 3.152 | up |
